# Semantic annotation for computational pathology: multidisciplinary experience and best practice recommendations

**DOI:** 10.1002/cjp2.256

**Published:** 2022-01-10

**Authors:** Noorul Wahab, Islam M Miligy, Katherine Dodd, Harvir Sahota, Michael Toss, Wenqi Lu, Mostafa Jahanifar, Mohsin Bilal, Simon Graham, Young Park, Giorgos Hadjigeorghiou, Abhir Bhalerao, Ayat G Lashen, Asmaa Y Ibrahim, Ayaka Katayama, Henry O Ebili, Matthew Parkin, Tom Sorell, Shan E Ahmed Raza, Emily Hero, Hesham Eldaly, Yee Wah Tsang, Kishore Gopalakrishnan, David Snead, Emad Rakha, Nasir Rajpoot, Fayyaz Minhas

**Affiliations:** ^1^ Tissue Image Analytics Centre University of Warwick Coventry UK; ^2^ Pathology University of Nottingham Nottingham UK; ^3^ Department of Pathology, Faculty of Medicine Menoufia University Shebin El‐Kom Egypt; ^4^ Histopathology University Hospital Coventry and Warwickshire Coventry UK; ^5^ Graduate School of Medicine Gunma University Maebashi Japan; ^6^ Department of Politics and International Studies University of Warwick Coventry UK; ^7^ Leicester Royal Infirmary, Histopathology University Hospitals Leicester Leicester UK

**Keywords:** whole‐slide images, computational pathology, annotations, guidelines

## Abstract

Recent advances in whole‐slide imaging (WSI) technology have led to the development of a myriad of computer vision and artificial intelligence‐based diagnostic, prognostic, and predictive algorithms. Computational Pathology (CPath) offers an integrated solution to utilise information embedded in pathology WSIs beyond what can be obtained through visual assessment. For automated analysis of WSIs and validation of machine learning (ML) models, annotations at the slide, tissue, and cellular levels are required. The annotation of important visual constructs in pathology images is an important component of CPath projects. Improper annotations can result in algorithms that are hard to interpret and can potentially produce inaccurate and inconsistent results. Despite the crucial role of annotations in CPath projects, there are no well‐defined guidelines or best practices on how annotations should be carried out. In this paper, we address this shortcoming by presenting the experience and best practices acquired during the execution of a large‐scale annotation exercise involving a multidisciplinary team of pathologists, ML experts, and researchers as part of the *Path*ology image data *L*ake for *A*nalytics, *K*nowledge and *E*ducation (PathLAKE) consortium. We present a real‐world case study along with examples of different types of annotations, diagnostic algorithm, annotation data dictionary, and annotation constructs. The analyses reported in this work highlight best practice recommendations that can be used as annotation guidelines over the lifecycle of a CPath project.

## Introduction

Recent developments in imaging technology, digitisation of glass slides, and artificial intelligence (AI) have spurred an ongoing revolution in clinical histopathology workflows and enabled automated analysis of digital pathology whole‐slide images (WSIs). This is evidenced by growth in commercial and government investment in Computational Pathology (CPath) as well as the rapid rise in the number of scientific publications in this field [1, 2]. In the United Kingdom, the *Path*ology image data *L*ake for *A*nalytics, *K*nowledge and *E*ducation (PathLAKE) consortium has been supported by £15 million fund to create a unique data resource of pathology images (a ‘data lake’) and develop AI technologies for cancer diagnosis and personalised treatment for routine clinical practice. Similar large‐scale CPath projects are underway elsewhere, such as the BIGPICTURE initiative [3].

**Figure 1:**
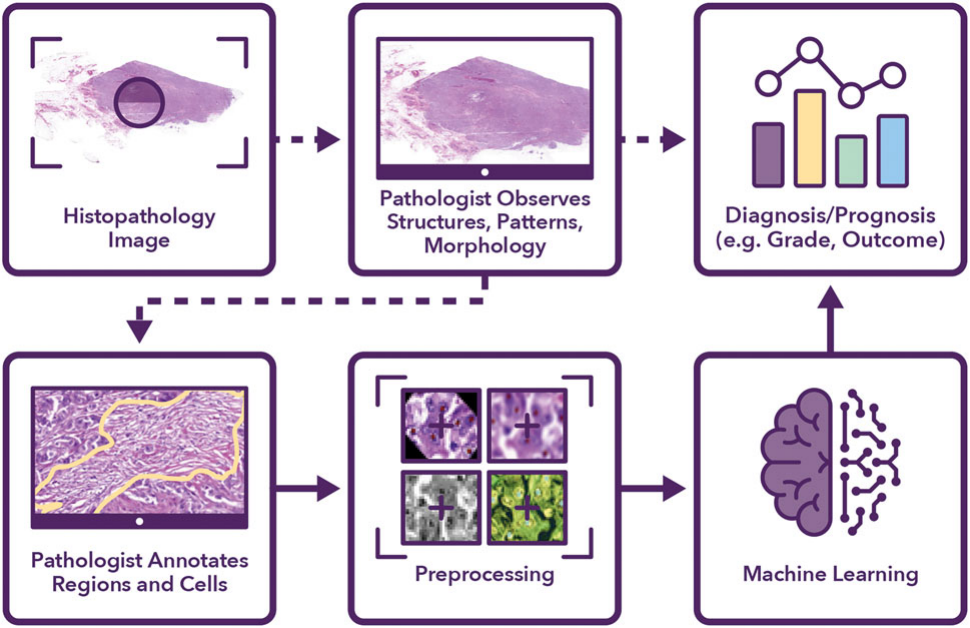
Manual versus automated process of histopathology image‐based diagnosis/prognosis. The dotted arrows show the manual process, whereas the solid arrows show the steps involved in automating the process.

CPath algorithms utilise the fact that there is fundamental information of diagnostic and prognostic benefit embedded in WSIs [4]. The typical lifecycle of a histological image analysis project in CPath is shown in Figure 1. Digitised tissue slides may be viewed online for remote consultation and can be processed by digital image processing and machine learning (ML) algorithms for the development of diagnostic and prognostic tools [5]. The ability of ML approaches to mine ‘sub‐visual’ image features in WSIs that may not be discernible to a pathologist can lead to improved quantitative modelling of disease characteristics and patient outcome [6] (supplementary material, Section S1.1).

The quality of annotation data and any biases in data collection, algorithm evaluation, biological and technical variations, and imaging quality can directly impact the efficacy of the resulting ML‐based CPath solution [7, 8]. A sufficient amount of well‐labelled/annotated/curated data is required to train ML models [9, 10]. With approaches such as self‐supervised learning, weak supervision, domain adaptation, and transfer learning, there has been significant progress in ML on using a small amount of annotated data for training algorithms that are robust and generalise well to unseen WSIs [11, 12, 13, 14, 15, 16, 17]. However, validation of even these label‐efficient algorithms and root cause analysis of failure cases of these algorithms still requires annotations. In addition to the requirement of annotations for training ML techniques, clinical deployment of these methods also warrants using well‐annotated samples for strong validation to ensure robustness and interpretability of ‘black‐box’ or ‘grey‐box’ AI models [18]. Generating these annotations is a labour‐intensive process because of the large volumes of data involved. Crowdsourcing may be cheaper and quicker but has the potential of introducing inconsistency, inaccuracies, and the difficulty of maintaining quality control (QC), as well as ethical issues of sharing clinically sensitive data [9].

There is no existing reference methodology for annotating different structures in WSIs for the development of ML approaches in CPath. The diversity of CPath solutions in terms of their objectives and diverse tissue types adds to challenges in defining a unified annotation protocol in CPath. Therefore, without any guidelines on how these annotations should be collected and used, there may be a significant repetition of effort across different CPath projects in quality assurance for making, managing, and using annotations. Standardisation of annotation and metadata storage along with imaging data is also an open problem in this domain. Possibilities for such standardisation include using DICOM [19] or the OME format which supports structured annotations. Similarly, initiatives towards sharing of standards, data, and methods will pave the way to collaborative CPath [20].

To address the above‐mentioned challenges in annotations for CPath projects, we propose an annotation workflow in this paper based on our experience in PathLAKE exemplar projects. We hope that these guidelines will pave the way for interoperability of annotation protocols, improved generalisability of algorithms via multicentre validation, and initiating a wider discussion on stringent annotation protocols in CPath.

## Materials and methods

In this section, we discuss our proposed workflow for semantic annotation of pathology images for CPath projects. The study was approved by the Yorkshire & The Humber – Leeds East Research Ethics Committee (REC Reference: 19/YH/0293) under the IRAS Project ID: 266925. Data collected were fully anonymised.

### Proposed annotation workflow

**Figure 2:**
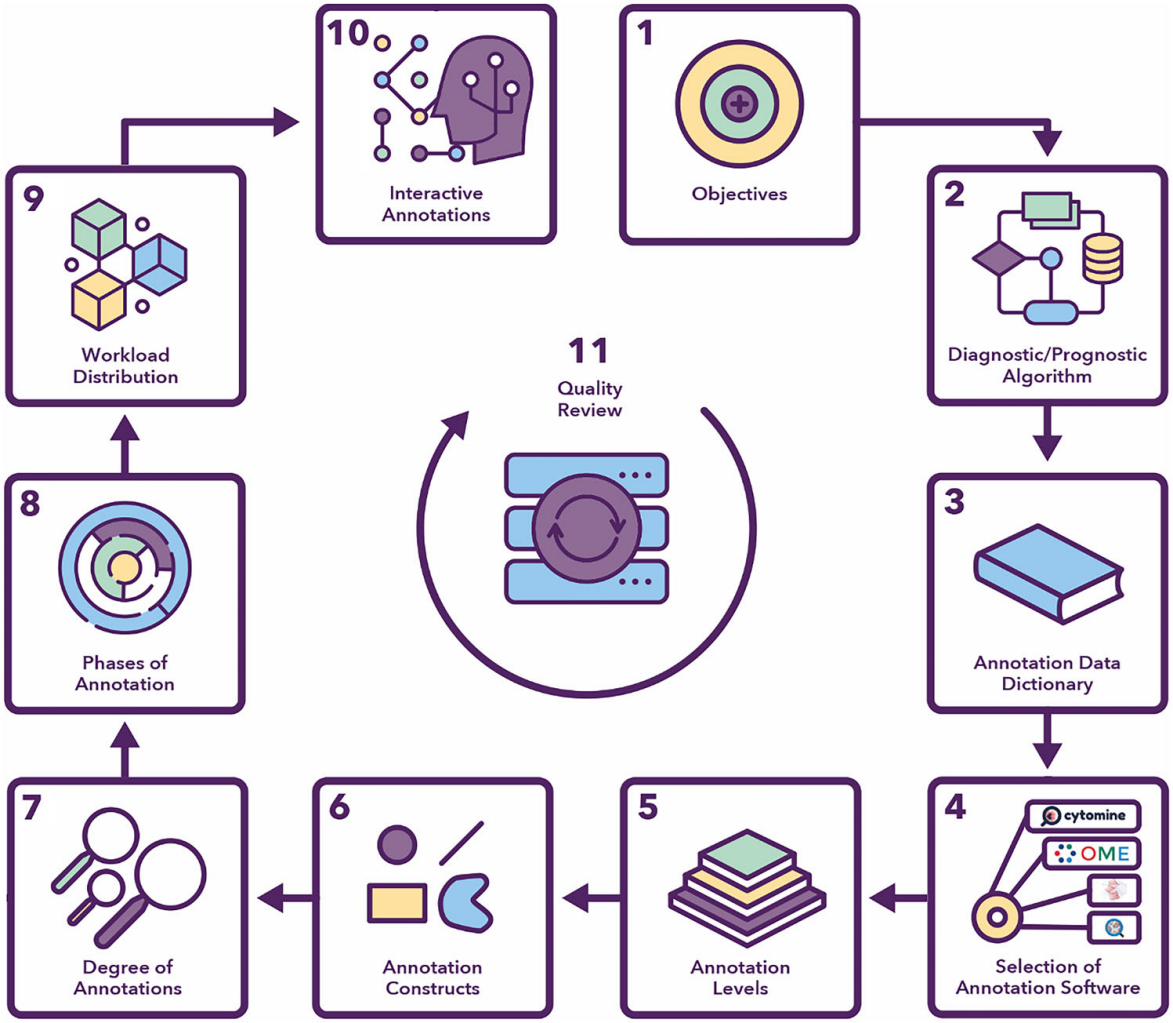
Proposed annotation workflow for a CPath project.

The proposed annotation workflow is illustrated in Figure 2 and each step is further discussed in the following sections.

### Definition of project objectives

The annotation process will be guided mainly by the specific objectives of the project. Therefore, we propose defining project objectives as the first step in the annotation workflow which will help align the annotation protocol with these objectives. For example, if the primary objective of a CPath project is to automate the process of grading breast cancer (BCa) in WSIs, then different structures relevant to the grading process, such as tubules, tumour cell morphology, and mitotic figures, should be part of the annotation protocol.

### Development of clinical diagnostic and/or prognostic algorithms

To identify the relevant clinical and diagnostic constructs for downstream ML solutions, we propose developing a clinical diagnostic/prognostic algorithm as the second step in the CPath project. Such algorithms enumerate steps that pathologists would perform for the routine diagnosis or prognosis of the disease or outcome of interest. The development of a clear and accurate clinical algorithm will guide the rest of the annotation workflow and ensure a clear understanding of the significant aspects of the problem by the multidisciplinary project team.

### Development of an annotation data dictionary

We propose the development of a *data dictionary* for every annotation project which is a standard reference document throughout the lifecycle of the project. Realisation of the diagnostic algorithm forms the basis of the data dictionary and defines different annotation constructs. The dictionary can serve multiple purposes. It can facilitate agreement on specific definitions of regions and cells and prevent concept drift over and beyond project lifetime. It can also act as a communication tool between pathologists, ML experts, and other collaborators. Furthermore, case‐level and WSI‐level labels in the annotation data dictionary can be associated with existing ontologies such as Systematized Nomenclature of Medicine (SNOMED) codes [21]. The data dictionary may include information to answer common project‐related questions, for example: What needs to be annotated? What is the diagnostic/prognostic value of each annotation type? What order to follow for annotations? How much to annotate (e.g. exhaustive, non‐exhaustive)? Clear examples of typical diagnostic cases with illustrative images facilitate the task of training new project staff. Parts of the PathLAKE data dictionary for our BCa exemplar project are provided in supplementary material, Section S3.1 (Figure S9 and Tables S2–S5).

### Selection of annotation software

Annotation of a WSI is a detailed and time‐consuming process for pathologists. Therefore, it is important to use a user‐friendly and easily accessible annotation software. The following factors should be considered when selecting an annotation tool: Does it support all annotation constructs defined for the project? Is it web‐based or desktop‐based? Does it have a workflow module, including the ability to configure a data dictionary and annotation style to all annotators for a CPath project (supplementary material, Table S5)? How does it store the annotations and the related meta‐data? How secure is the system? Some more factors are mentioned in supplementary material, Section S2.1.

There are several open‐source tools available for annotating histopathology images (supplementary material, Table S1) and these are briefly discussed in supplementary material, Section S2.1. A description of steps involved in doing the annotations using the selected software and defined data dictionary can be found in supplementary material, Figure S1. These steps are documented in a proper standard operating procedure (SOP) so that everyone, especially new members, can easily follow the flow of making annotations.

### Defining annotation levels

**Figure 3:**
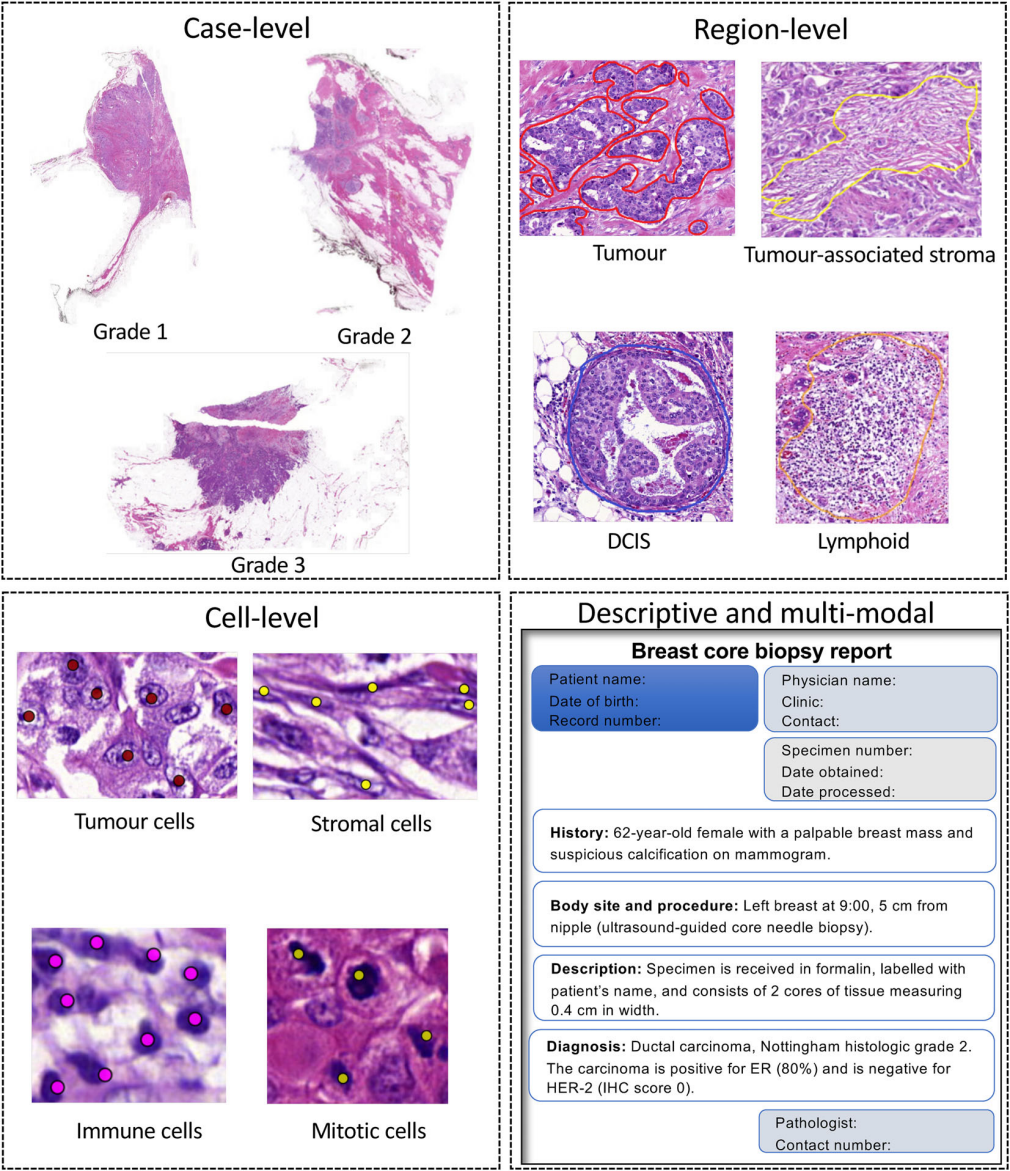
The four proposed levels of annotation.

For achieving the aims and objectives of an ML project, annotations should be marked at different levels of detail. For example, keeping the case/slide‐level annotations at the first level can make the computational analysis efficient since it is less time‐consuming than marking annotation constructs at region‐ and cell‐level. A more detailed level analysis, which may use more explainable features, will require further detailed annotations of the individual WSI. Descriptive and multi‐modal annotations could also be considered to exploit the information stored in the form of pathology reports and other associated genomic and transcriptomics. Figure 3 shows the four levels of annotations. Further details on the levels of annotations are provided in supplementary material, Section S2.2.

### Defining annotation constructs

Different annotation shapes can be used for different types and levels of annotations. The main annotation constructs include bounding box, point/circle, polygon, line, and text (supplementary material, Section S2.3 and Figure S4). Different line‐style, linewidth, line‐colour, fill‐colour, and so on can be defined in the annotation protocol to use the same constructs for further categorisation (supplementary material, Table S5).

### Degree of annotation

Annotations of CPath WSIs can be performed at varying degrees of exhaustiveness. In exhaustive annotations, all the features that exist inside a construct (such as a freehand polygon, bounding box, or even the entire WSI) are annotated. Such annotations make the evaluation of an ML model easier. The required number of cell‐level and region‐level boxes should be defined so that each WSI could be checked for completeness of the annotations. In non‐exhaustive annotations, regions and cells of interest in different areas of the image are marked in a non‐exhaustive manner (supplementary material, Figure S5). Depending on a project's objective, another important aspect is the estimation of sample size [22, 23]. A large sample size may be required when developing a prognostic algorithm where the objective is to measure very minute differences between groups.

### Phases of annotation

We recommend that annotations be carried out in a phased manner where each phase focuses on a particular level of annotations (supplementary material, Figure S6). A pilot phase will help in early identification and possible avoidance of problems in later phases of the project. It can also help train the annotation team with new constructs and terms defined in the data dictionary. In the first phase, the slides are assigned a case‐ or slide‐level label. Case‐level annotations can be used in a weakly supervised manner for building ML models. Depending on the nature of problem, the second phase can either be region‐level or cell‐level annotations.

### Interactive and active annotations

When the annotation budget is small or there is a limited availability of annotation experts, interactive annotation and active learning can be used to speed up exhaustive annotations (supplementary material, Section S2.6). In interactive annotation, the user reviews the output of the annotation model and provides feedback to improve model's performance. Active learning works in an iterative manner where the annotation model asks the user (teacher) for samples from an unannotated data set such that the performance of the model improves.

### Workload distribution

It is not trivial to accurately estimate workload distribution because of the complex nature of histopathology image annotation, involving different levels (cases, regions, cells, descriptive reports), details (exhaustiveness, concordance), and pathologists' experience, clinical time constraints, and daily work commitments [24]. A better distribution of workload can be arrived at by listing the number of cases to annotate, the number and types of annotations per phase, timeframe, and the number of available pathologists. A pilot phase or initial analysis of the annotation might be helpful in the workload estimation. Similarly, an annotation tool facilitating the automatic assignment of annotation tasks can ease assigning cases to pathologists for annotation but, to the best of our knowledge, there is no such tool publicly available.

### Quality review

Quantitative analysis relies on the quality of the WSIs which in turn depends on the quality of tissue sectioning, staining, and scanning. Supplementary material, Section S2.7 describes how these steps can affect the annotations and hence the ML‐based analysis.

#### QC of images

The staining and scanning quality of images is important for good annotations and hence better ML models. Only images passing ImageQC [25] should be included for further processing and analysis. In PathLAKE at Warwick, we have used an in‐house reproducible and automated image quality analysis pipeline (ImageQC, supplementary material, Section S2.8) for precisely localised artefacts to identify slides that need to be re‐scanned or regions that should be avoided during computational analysis. Other software can also be used for ImageQC such as HistoQC and PathProfiler [25, 26].

#### Annotation quality

During the whole process of annotation, annotation quality should be regularly reviewed. This can help annotators identify their errors and improve the annotation quality over time.

#### QC metrics for annotation

For a detailed QC of annotations, we propose four metrics to measure completeness, exhaustiveness, diversity, and agreement of annotations (Table 1). For the annotation data dictionary, ‘completeness’ criterion ensures that the annotations for an image are complete in terms of the required number of cell‐level and region‐level boxes by the required number of annotators. The exhaustiveness criterion makes sure that all the structures (regions, cells, etc) in a region‐box are annotated as much as possible. To obtain a sufficient percentage of annotated regions, some basic tissue segmentation/thresholding is required so that the non‐tissue area is discarded in the calculations. Based on some initial annotations, a threshold can then be defined to identify cases not satisfying the exhaustiveness criterion. Similarly, the agreement criterion measures the agreement between multiple annotators. Different metrics can be used to measure inter‐annotator agreement (see supplementary material, Section S2.9 for further details).

**Table 1:** Proposed annotation QC metrics.

Matric name	Purpose	Unit
Completeness	Are the annotations complete according to the defined protocol?	Yes/no
Exhaustiveness	What percentage of tissue is annotated in the defined box(es)?	Percentage area
Diversity	How many types of regions are annotated?	1 to number of defined types in the protocol
Agreement	How much the annotators agree on regions? How much the annotators agree on cells?	Jaccard similarity index Cohen's kappa

##### Automatic QC of annotations

**Figure 4:**
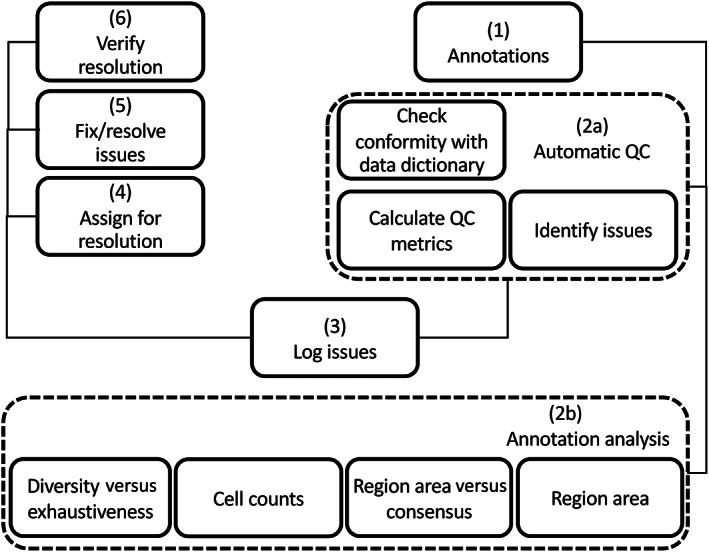
Proposed annotation QC steps.

Manual review of all the annotations is a time‐consuming task. For large CPath projects, an automatic QC pipeline is required to identify problematic annotations. Depending on the types of annotations, different QC steps can be defined. Figure 4 shows generic steps involved in the automatic QC and analysis of the annotations. Automatic QC of annotations checks if the annotations conform with the data dictionary, identifies issues, and calculates QC metrics such as exhaustiveness and concordance. Any issues identified are logged in the system with a unique annotation ID, WSI ID, logged date, and description of the issue for further triaging, assignment, and resolution. We recommend a regular review of the annotation by pathologists (supplementary material, Section S2.10). The calculated QC metrics can then be used for further analysis of the annotation, for example, to prioritise regions/cells based on the current area/count.

##### Annotation interoperability

For annotations to be interoperable with other software tools, there should a proper schema defined for all the styles and structures so that there is minimal overhead for translation for use with other systems. Annotation schema help standardise annotations and smooth the conversion process if the project involves annotations from multiple centres using different annotation software.

## Results

### Application of proposed guidelines in PathLAKE

In this section, we present the results of applying the guidelines discussed above for the Breast Cancer (BraCe) project under the PathLAKE consortium so that these can be used as a guide for future projects. The cases and the corresponding annotations for BraCe were collected from Nottingham City Hospital UK where seven pathologists (IMM, MT, AGL, AYI, AK, HOE, and MP) were involved in the annotation process.

### Project objectives

The objectives of the BraCe project were clearly defined in a project document and included in the data dictionary. The main objective of the BraCe project is ‘automatic analysis of breast cancer WSIs for grading and prognosis’.

### Diagnostic/prognostic algorithm

**Figure 5:**
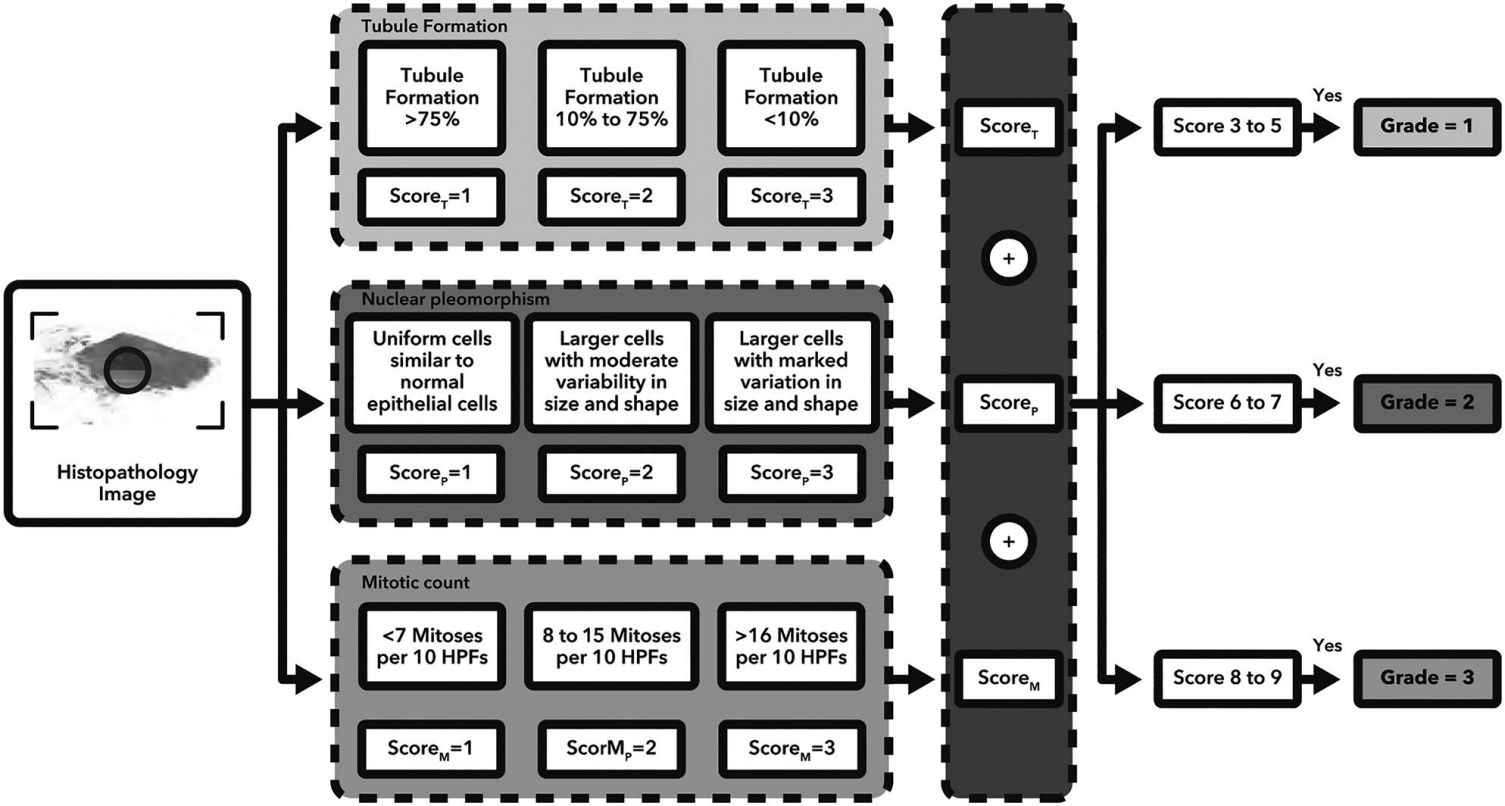
A diagnostic algorithm for assigning a grade to a breast cancer histopathology image. T, tubule formation; P, nuclear pleomorphism; M, mitotic count; HPF, high‐power field.

In line with the project objective, a clear and detailed clinical diagnostic algorithm for BCa grading was specified in Figure 5. The algorithm provides a holistic view of the different steps involved in the assessment process. In BCa grading, three main features (tubule formation, nuclear pleomorphism [NP], and mitotic count) are identified to calculate and assign a grade to each case [27].

### Annotation: data dictionary, software, levels, and constructs

The main parts of the BraCe annotation data dictionary are provided in Figure S9 (types of bounding boxes) and Tables S2–S5 (description of boxes, regions, and cells) described in supplementary material, Section S3.1. An open‐source online annotation software [28] was customised for making annotations (supplementary material, Section S3.2). Annotations were saved in JSON format with fields for slide ID, annotator ID, stain type (H&E, IHC), feature type (region, cell), and feature name (tumour, stroma, etc). Annotations were conducted at case‐level, region‐level, and cell‐level, as detailed in supplementary material, Tables S6–S12. Supplementary material, Figures S2 and S3 show sample annotations. Further details of annotation collection with timeline are provided in supplementary material, Section S3.3. Supplementary material, Figure S10A shows a timeline of the overall region‐ and cell‐level annotations. Similarly, supplementary material, Figures S10B and S10C present details of the different types of region‐level and cell‐level annotations, respectively. In total, 10,731 bounding boxes; 509,591 cells; and 194,717 regions were annotated (supplementary material, Section S3.4).

### Degree and phases of annotation

All the annotations were carried out in an exhaustive manner and were quantified with the proposed exhaustiveness metric further detailed in supplementary material, Section S3.5. Supplementary material, Figure S11 shows the comparison of the exhaustiveness versus diversity of H&E region‐level annotations for individual and consensus boxes for a subset of 40 cases. For familiarity with the definitions and structures, two multi‐disciplinary workshops were held where a small set of representative cases was used. Following a pilot phase, relevant regions and cells were annotated in a phased manner (supplementary material, Section S3.6).

### Interactive/active annotations and workload distribution

Interactive annotations of about 124,624 H&E and 109,862 progesterone receptor (PR)‐stained cell boundaries were generated using NuClick [16] (supplementary material, Section S3.7). Supplementary material, Figure S7 shows some nuclei boundary segmentation for different types of cells. Workload distribution was estimated based on the pilot phase and initial annotations (supplementary material, Section S3.8). A mix of pathologist experience was ensured in each team of pathologists.

### Quality review

#### Image/annotation quality analysis and pathologists' agreement

The results of ImageQC pipeline (supplementary material, Section S3.9) on WSIs with pen‐marking, coverslip edges, and blurriness are shown in supplementary material, Figure S12. The bar chart in supplementary material, Figure S13 shows the annotations of different types of regions in terms of counts and area (in mm^2^). This type of analysis helps in prioritising the regions for annotations. Similarly, the inter‐annotator agreement for sample regions in terms of Jaccard similarity index (JSI) versus the area of the regions are shown in supplementary material, Figure S14. Slides were assigned in groups of two pathologists for measuring inter‐annotator agreement. As it is often quite difficult to delineate the exact boundaries of different regions, the agreement, in terms of JSI, among pathologists is also low. For example, a good amount of area (about 28 mm^2^) is annotated for tumour‐associated stroma but JSI is still quite low (about 0.44) as compared to the ideal index of 1 (supplementary material, Figure S14).

**Figure 6:**
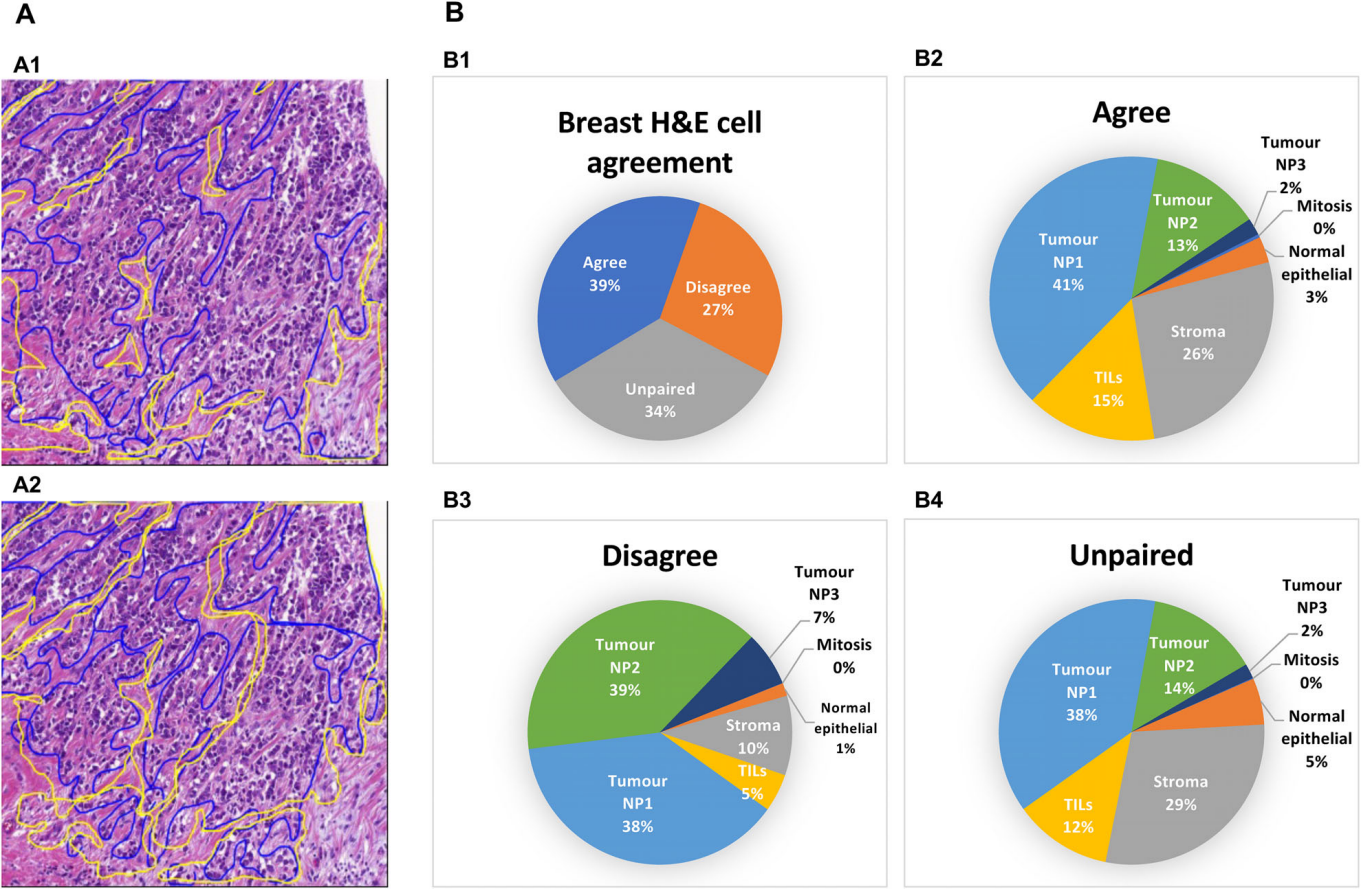
(A) An example of annotation variability between two pathologists (A1, tumour‐associated stroma; A2, tumour). Annotations by pathologist 1 (blue) and pathologist 2 (yellow). (B) Mean percentage of cells (B1) on which two pathologists agreed (B2), disagreed (B3), and missed by one pathologist (B4) in breast H&E cell annotations.

Figure 6A shows examples of variability among pathologists for two region types of tumour and tumour‐associated stroma. Inter‐pathologist variability might be a result of annotator's bias, experience, judgement, ambiguous definitions in the data dictionary, or the difficulty in delineating some regions.

Inter‐pathologist agreement/disagreement on cell‐level annotations is shown in Figure 6B. To measure the agreement, point annotations within a radius of 12 pixels at ×40 magnification (approximately 0.25 μm per pixel) were considered as annotation for the same cell. It can be observed that it was quite common for the pathologists to miss some cells (34%, Figure 6B1), even when exhaustively annotating inside a bounding box. Specific types of cell nuclei annotated by one pathologist, but missed by another, include mostly tumour NP1 and NP2, stroma, and tumour‐infiltrating lymphocytes (TILs) (Figure 6B4). The highest disagreement is exhibited for tumour cells pleomorphism (Figure 6B3). It is important to discuss such issues of disagreement in pathologists' review meetings to reach a consensus (e.g. discussing and updating the features of pleomorphism). The weighted Cohen's kappa on cells annotated by two annotators was 0.77 (when tumour cells were categorised into NP1, 2, and 3) and 0.80 (without tumour cells categorisation).

To further analyse the inter‐pathologist discordance on different cell types, supplementary material, Table S14 presents the confusion matrix. It is evident that the different categories of NP are quite challenging to identify, especially differentiating NP1 from NP2 (4,699 disagreed).

#### Annotation usage

As a demonstrative example, we present our results on using the annotations gathered in our BraCe project for the development of an ML model for classification of different breast cells in H&E WSIs. For this purpose, our in‐house HoVerNet cell classification and segmentation model [29], which was pre‐trained on the PanNuke data set [30], was fine‐tuned to classify breast H&E cells by using a subset of the annotation in a three‐fold cross‐validation protocol (supplementary material, Section S3.9.4). The number of cell annotation included 20,822 tumours; 3,134 TILs; 7,119 stromal; and 1,528 normal epithelial cells. A marked improvement in classification results, from a macro F1 score of 0.53 to 0.79 (supplementary material, Table S15), with the use of the annotations clearly demonstrating the usefulness of annotations collected in this manner.

## Discussion

To increase the usage of annotated data sets, it is important that a standard annotation protocol agreed between pathologists and the ML team is followed. In the absence of a standard protocol, it can become very difficult to make use of the existing annotated data sets. Similarly, interoperability of annotations is a big issue currently. For example, because of the existence of many different image formats and compression models for storing WSIs, an annotation software might not support all the available file formats for different scanners. Furthermore, each annotation software has its own unique way of storing the annotations (XML, JSON, CSV) and then a conversion process will be required. To make use of an annotated data set, one should ideally only require the annotation file and its corresponding data dictionary. There is also a need to standardise the process of annotation reviews by the pathologists so that discordant annotations can be resolved in a systematic manner.

It is important to note that the annotation strategy should be determined by the task, and this would mainly be determined by discussion between the ML and pathology teams. Other approaches of annotations might be considered if conventional cell‐level or region‐level annotations are not sufficient for making an assessment [31].

To the best of our knowledge, no other guidelines for CPath annotations are available in the public domain. The proposed guidelines can form the basis for a community‐wide consensus and refinement of the annotation process as well as standardisation of annotation storage and sharing. Below, we discuss some limitations in CPath annotations and other annotation strategies and list some recommendations based on our experience.

### Limitations and other annotation strategies

As mentioned earlier, annotations by histopathologists, ideally a sub‐specialist in the domain of CPath projects, trained on the annotation system and the designed protocol are very important for training and validation of ML methods especially when a new tissue or cell type is being explored. However, some annotations could be performed by trainees and other human experts (such as crowdsourcing) including easily differentiated regions such as fat, ductal carcinoma *in situ*, and so on, which can then be verified by a pathologist [9]. Cell‐level annotations could be difficult to collect from non‐pathologists because the verification process itself may be time‐consuming.

The manual annotations performed by pathologists are known to be potentially subjective [32]. Therefore, concordance of annotations where more than one pathologist is involved may be an issue as reported in the Results section. Similarly, getting annotations from expert pathologists is time‐consuming as well as costly. Furthermore, the complexities of the annotation tool used and the level of training required to become familiar with the tool will add to the time cost of manual annotations.

Complementary annotation strategies include the use of interactive annotations, unsupervised, and semantic segmentation approaches, as well as weakly supervised methods [33, 34]. Interactive annotations start with some manual annotations to train an AI model which can then help in generating automatic annotations. These interactive annotations can then be confirmed or edited by experts. The main limitation of this strategy is that it may sometimes take more time to amend the automatic annotations than to make new ones. Similarly, in AI‐assisted annotations, an unsupervised or weakly supervised AI model generates the annotations for manual verification by experts. However, the verification may be time‐consuming and the AI annotations may bias the pathologist to agree with the AI annotations on regions or cells that are hard for humans to differentiate [35]. In ML, there is a relatively new learning method called zero‐shot learning, but to the best of our knowledge there is no existing work on this learning strategy for unsupervised CPath annotations.

### Recommendations

In this section, we make some recommendations to address some of the above‐mentioned limitations.

- Prior to making the annotations, a diagnostic/prognostic algorithm should be designed so that both the pathologist and ML team are aware of the main goal and the purpose of the annotations.
- A CPath project team may find a pilot phase of annotation beneficial to identify issues regarding the usability of annotation tools, understanding of the data dictionary, and level of agreement on constructs, regions, and cells.
- To allow a variety of downstream ML analyses, a large number of region types can be allowed for region‐level annotations initially and merged later, if necessary.
- Using some of the initial annotations by an ML algorithm will help identify difficult regions and cells which can then be prioritised for annotation.
- Inter‐annotator discordance should be discussed in regular pathologist meetings to reach an agreement or clarification of terms in the data dictionary for unambiguous definitions.
- In case of ambiguous structures, where the pathologist is not sure about the category, it is advisable to keep a category of ‘unknown’ regions and cells to avoid noisy annotations for ML model training and further assessment.

In a future study, it would be useful to apply the proposed guidelines to a complete lifecycle of a CPath project to see the effects of some aspects, such as interactive and active annotations for expediting the annotation process, interoperability, and use of non‐exhaustive annotations. Similarly, the extension of the data dictionary and the associated annotation schema to other projects is required to see what overhead may be incurred in adaptation of the proposed annotation protocols.

## Author contributions statement

NR and FM conceived the study. NW carried out the experiments. IMM, MT, AGL, AYI, HOE, MP and AK provided annotations. AB, SEAR, MT, DS and ER developed the data dictionary. WL, MB and SG provided Image QC. MJ contributed the interactive annotation section. YP and GH developed the software. NR, FM, NW, IMM, KD and HS wrote and edited the paper. AB, MT, TS, EH, HE, YWT and KG reviewed the paper. All authors read and approved the final paper.

## Supporting information


Glossary of Machine Learning terms
Section S1. Introduction
S1.1 Advantages of machine learning in computational pathology (CPath)
Section S2. Materials and Methods
S2.1 Annotation software
S2.2 Levels of annotation
S2.3 Annotation constructs
S2.4 Degree of annotation
S2.5 Phases of annotation
S2.6 Interactive and active annotations
S2.7 Quality of tissue sectioning, staining and scanning
S2.8 Image quality control
S2.9 Measuring inter‐annotator agreement
S2.10 Pathologists' review of annotation
Section S3. Results (Applied example)
S3.1 Annotation data dictionary
S3.2 Annotation software
S3.3 Annotation levels
S3.4 Annotation constructs
S3.5 Degree of annotation
S3.6 Phases of annotation
S3.7 Interactive/active annotations
S3.8 Workload distribution
S3.9 Quality review

## Data Availability

All annotations and the corresponding annotation protocols will be made available upon completion of the PathLAKE project.
