# Supplementary material for: Semantic annotation for computational pathology: multidisciplinary experience and best practice recommendations
Source: J Pathol Clin Res. 2022 Jan 10;8(2):116–28. doi: 10.1002/cjp2.256 (PMC8822374; doi:10.1002/cjp2.256)
Supplement: Supplementary file 1 — Glossary of Machine Learning terms Section S1. Introduction S1.1 Advantages of machine learning in computational pathology (CPath) Section S2. Materials and Methods S2.1 Annotation software S2.2 Levels of annotation S2.3 Annotation constructs S2.4 Degree of annotation S2.5 Phases of annotation S2.6 Interactive and active annotations S2.7 Quality of tissue sectioning, staining and scanning S2.8 Image quality control S2.9 Measuring inter‐annotator agreement S2.10 Pathologists' review of annotation Section S3. Results (Applied example) S3.1 Annotation data dictionary S3.2 Annotation software S3.3 Annotation levels S3.4 Annotation constructs S3.5 Degree of annotation S3.6 Phases of annotation S3.7 Interactive/active annotations S3.8 Workload distribution S3.9 Quality review [file CJP2-8-116-s001.docx]

**Semantic annotation for computational pathology: multidisciplinary experience and best practice recommendations**

N Wahab *et al*. *J Pathol Clin Res* DOI: 10.1002.cjp2.256

**Supplementary Material**

*Reference numbers refer to the main text list.

**Table of Contents**

[**Glossary of Machine Learning terms** 3](#_Toc89268877)

[**Section S1 - Introduction** 5](#_Toc89268878)

[**S1.1 Advantages of machine learning in computational pathology (CPath)** 5](#_Toc89268879)

[**Section S2 - Materials and Methods** 5](#_Toc89268880)

[**S2.1 Annotation software** 5](#_Toc89268881)

[**S2.2 Levels of annotation** 10](#_Toc89268882)

[**S2.2.1 Case-level annotations** 10](#_Toc89268883)

[**S2.2.2 Region-level annotations** 10](#_Toc89268884)

[**S2.2.3 Cell-level annotations** 12](#_Toc89268885)

[**S2.2.4 Descriptive and multi-modal annotations** 13](#_Toc89268886)

[**S2.3 Annotation constructs** 13](#_Toc89268887)

[**S2.4 Degree of annotation** 15](#_Toc89268888)

[**S2.5 Phases of annotation** 16](#_Toc89268889)

[**S2.6 Interactive and active annotations** 17](#_Toc89268890)

[**S2.7 Quality of tissue sectioning, staining and scanning** 18](#_Toc89268891)

[**S2.7.1 Tissue sectioning quality** 18](#_Toc89268892)

[**S2.7.2 Staining quality** 19](#_Toc89268893)

[**S2.7.3 Scanning quality** 19](#_Toc89268894)

[**S2.8 Image quality control** 19](#_Toc89268895)

[**S2.9 Measuring inter-annotator agreement** 21](#_Toc89268896)

[**S2.10 Pathologists’ review of annotation** 22](#_Toc89268897)

[**Section S3. Results (Applied example)** 23](#_Toc89268898)

[**S3.1 Annotation data dictionary** 23](#_Toc89268899)

[**S3.2 Annotation software** 37](#_Toc89268900)

[**S3.3 Annotation levels** 37](#_Toc89268901)

[**S3.4 Annotation constructs** 48](#_Toc89268902)

[**S3.5 Degree of annotation** 50](#_Toc89268903)

[**S3.6 Phases of annotation** 51](#_Toc89268904)

[**S3.7 Interactive/active annotations** 52](#_Toc89268905)

[**S3.8 Workload distribution** 53](#_Toc89268906)

[**S3.9 Quality review** 54](#_Toc89268907)

[**S3.9.1 Image Quality analysis** 54](#_Toc89268908)

[**S3.9.2 Annotation Quality analysis** 56](#_Toc89268909)

[**S3.9.3 Pathologist agreement** 59](#_Toc89268910)

[**S3.9.4 Annotation usage** 60](#_Toc89268911)

# **Glossary of Machine Learning terms**

| **Term** | **Description** |
| --- | --- |
| Computational pathology | Using Artificial Intelligence for constructing diagnostic, predictive and prognostic models by incorporating multiple sources of data (pathology imaging, molecular markers, sequencing, etc.) |
| Artificial Intelligence | A program or model whose purpose is to reproduce the cognitive abilities of a human being. |
| Algorithm | A set of rules to perform a task or to solve a problem. (e.g., classification, regression, and clustering algorithms) |
| Black-box model | It refers to a model whose decision is hard to explain in connection to the data features it used. |
| Grey-box model | A model that has a partial theoretical structure along with data to produce more interpretable and explainable outputs in comparison to black-box models |
| Training | Determining the optimal parameters for a model |
| Inference | Using a trained model to make predictions on unlabelled or test examples. |
| Supervised learning | A training approach which requires annotated/labelled examples. |
| Self-supervised learning | A training approach which uses pseudo labels from unlabelled examples for training. |
| Transfer Learning | Transfer of information from one machine learning task to another. |
| Weakly-supervised learning | A training approach which uses limited or imprecise training labels in contrast to precise labels used in fully-supervised learning. |
| Active learning | A machine learning paradigm in which a user can be interactively queried to label new data points which are then used for training. |

# **Section S1 - Introduction**

## **S1.1 Advantages of machine learning in computational pathology (CPath)**

Using ML in CPath can help overcome major challenges in clinical practice such as data or material sharing, subjectivity [*36], non-reproducibility due to inter- and intra- observer disagreement [*32], inefficiency in the form of delayed and inaccurate diagnoses [*37] and integration with genomic or multidomain data [*38, *39]. With an ever-increasing number of patients, a global decrease in doctors enrolling to the pathology specialty and an ageing workforce [*40, *41], time and cost efficiency in clinical pathology is of utmost importance. AI methods can help reduce diagnosis times by automating the process or assisting pathologists in various diagnostic steps such as detecting regions of interest, highlighting different tissue types, counting cells, visualising clinically important structures and features, lymph node assessment for metastasis, and analysing a WSI for grading [*42].

# **Section S2 - Materials and Methods**

## **S2.1 Annotation software**

Selecting an appropriate annotation software is important for making the annotations. This choice is usually based on the nature of annotations, size of the team, and duration of the project. Some factors (other than those discussed in the main text) that should be considered when selecting the annotation software: Is it easy enough to be used by the annotators or does it require extensive training? Does it support different image types/formats? Can it search by case or annotation type? Can it be upgraded securely to add extra functionalities without losing the work that has been already done before on it? Does it support adding annotation completeness criteria?

Table S1 compares different annotation software in terms of some main factors that are important for making the annotation process easy. All the listed software support basic annotation constructs such as drawing lines, circles/dots, rectangles and polygons. AIDA supports free-hand paths using a pen or pencil tool. Similarly, AIDA, Cytomine, DSA, and Omero can be accessed via the Internet but ASAP and QuPath are desktop based applications. Different software support different image formats which should be considered when working with some specific scanners or to consider file conversions. Cytomine, DSA, Omero and QuPath have some ways of integrating ML results as well. Feature of viewing two or more versions of the same image (for example, image with pathologist annotation and image with ML results) side-by-side is very useful for a collaborative discussion among ML experts and pathologists. Two (i.e., Cytomine and Omero) of the five listed software support such a feature. As all these software store the annotations in either JSON or XML format so upgrading the software would not affect already completed annotations. DSA provides group level security where the members of the same group can view annotations within the group but only the admin or the creator can edit or delete the annotations. ASAP and QuPath are desktop versions and the administrator can create accounts if access is required via a server. For uniform annotations, we recommend that the workflow of the actual annotation process including launching the software, logging in, selection of images and selection of annotation tool should also be documented as a flowchart. A sample flowchart for region-level annotation using the selected software (WASABI, further described in S3.2) is shown in Figure S1.

**Table S1** Comparison of different annotation software

| Software | 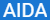AIDA [*43] | 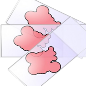ASAP [*44] | 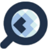Cytomine [*45] | 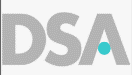DSA [28] | 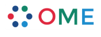Omero [*46] | 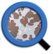QuPath [*47] |
| --- | --- | --- | --- | --- | --- | --- |
| Supported Annotation Constructs | Line, dot, rectangle, polygon, path, pencil, paint | Line, dot, rectangle, polygon, spline | Dot, rectangle, polygon, line, circle | Line, dot, rectangle, polygon | Line, dot, rectangle, polygon, text | Line, dot, rectangle, polygon |
| Internet accessibility | 🗸 | 🗴 | 🗸 | 🗸 | 🗸 | 🗴 |
| Supported Image formats | dzi | OpenSlide supported format (ndpi, svs, tif, mrxs, vms etc.) | mrxs, tif, tiff, vms, vsi, ndpi, svs, bif | mrxs, tiff, ndpi, svs | Bio-Formats (svs, h5, ndpi, tif, jp2, dicom etc.) | OpenSlide and Bio-Formats |
| Annotation format | JSON | XML | JSON | JSON | JSON | JSON |
| Open source | 🗸 | 🗸 | 🗸 | 🗸 | 🗸 | 🗸 |
| ML integration | 🗴 | 🗴 | 🗸 | 🗸 | Via third-party software | 🗸 |
| Overlay visualisation | 🗴 | 🗸 | 🗸 | 🗴 | 🗸 | 🗸 |
| Side-by-side viewing | 🗴 | 🗴 | 🗸 | 🗴 | 🗸 | 🗴 |


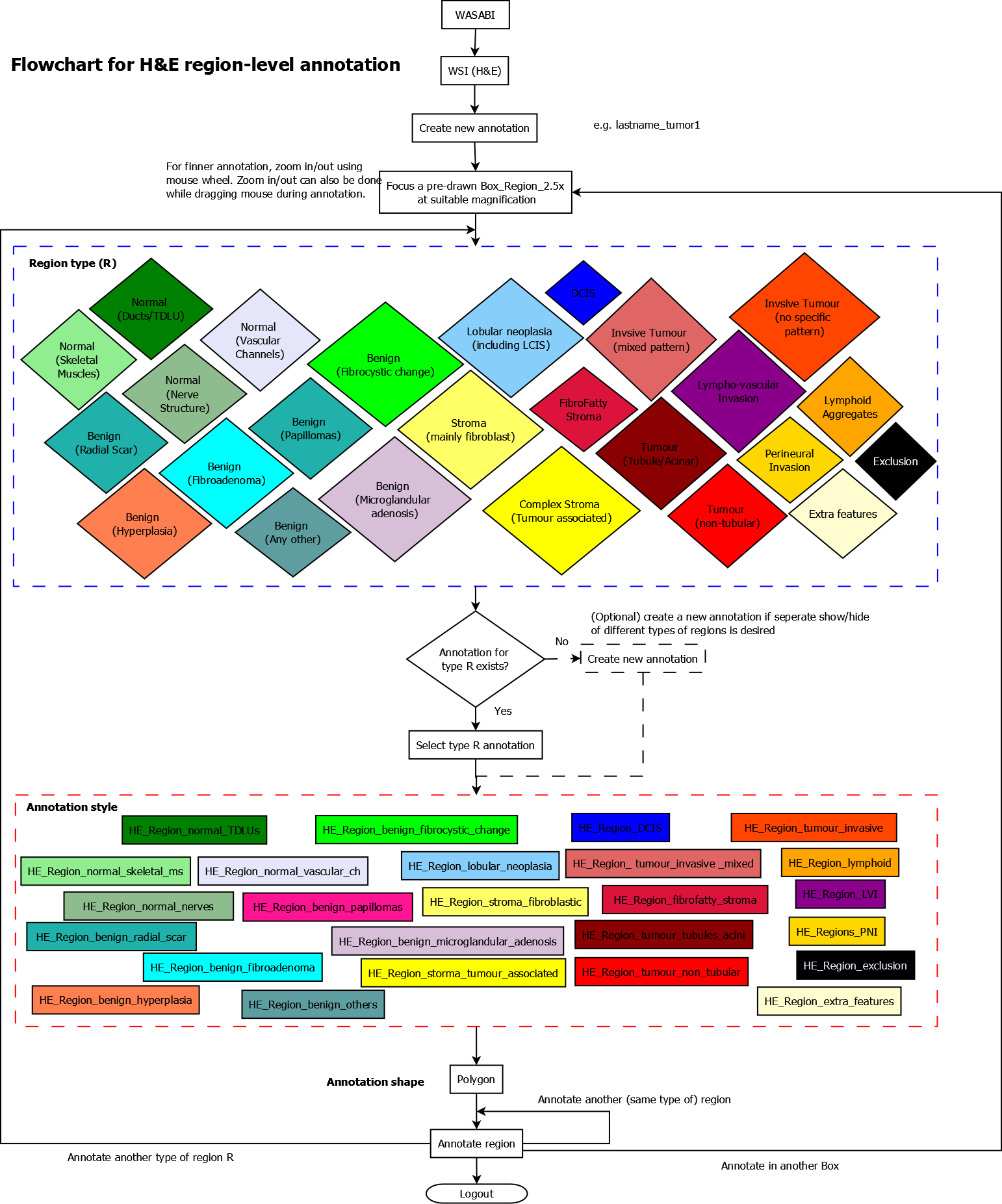


**Figure S1.** Flowchart for breast H&E region-level annotations and workflow of using the annotation software (WASABI, further described in S3.2)

## **S2.2 Levels of annotation**

At case-level a single or multiple (in case of multi-label problem) labels can be assigned to a case, for example, benign vs malignant. Region level annotations include different regions in the WSI which can be of any diagnostic or prognostic value for a project at hand, for example, tumour, stromal and ductal carcinoma in-situ (DCIS) regions in breast cancer. Cell-level annotations are the more detailed annotations in which different cell types are annotated. Descriptive and multi-modal annotations include pathology reports and genomics and transcriptomics.

### **S2.2.1 Case-level annotations**

Depending on the underlying task, a single or multiple label(s) can be assigned to a case and its representative slides or to each slide separately. The label can be binary (such as benign vs malignant) or multi-class (e.g., grade 1, 2, 3). These labels represent the overall diagnosis or prognosis. For example, if the case is grade 1, 2 or 3, or if it belongs to a high or low risk group. Such annotations can be used by ML models which do not require detailed annotations, known as weakly-supervised ML models. Deep convolutional neural network can be used as weakly-supervised classifier to extract useful features from the raw image data. For this purpose, the image is usually divided into multiple tiles for processing and aggregating the results to make a case-level prediction [*48]. In such a scenario, multiple instance learning (MIL) or other methods of aggregation can be used [*49–*51].

### **S2.2.2 Region-level annotations**

Region level annotations include different regions in the WSI which can be of any diagnostic or prognostic value for a project at hand, for example, tumour, stromal and ductal carcinoma in-situ (DCIS) regions in breast cancer. Figure S2 shows examples of region-level annotations. For flexibility in the degree of granularity in the downstream analysis, a large number of region types can be allowed for region-level annotations initially and merged later, if necessary. For example, invasive tumour region in BCa images can be differentiated into tubular, non-tubular, and mucinous regions. Region-level annotations can be used for training region segmentation models as well as restricting the detailed analysis to a specific area, for example, calculating the percentage of positive invasive tumour cells out of all invasive tumour cells for nuclear staining to Ki-67. Similarly, stromal region segmentation is required to obtain the percentage of tumour-infiltrating lymphocytes (TILs). Depending on ML needs, patches of different sizes can be extracted from the annotated regions for training/validating ML models. Region-level annotations are usually carried out by drawing polygons around the areas, but bounding boxes can similarly be used.


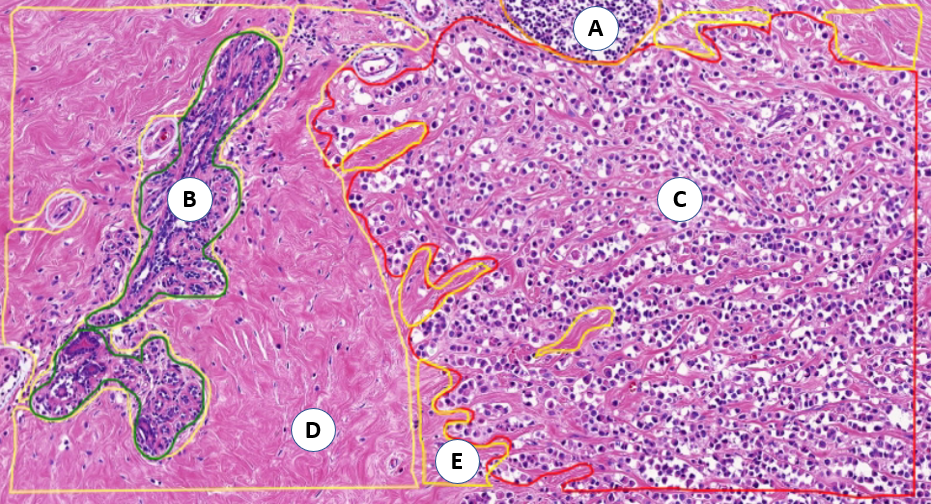


**Figure S2.** Region-level annotations. A) lymphoid aggregate, B) Normal terminal ductal lobular unit (TDLU), C) tumour, D) Stromal Fibroblast, E) Tumour-associated stroma

### **S2.2.3 Cell-level annotations**

Cell-level annotations are the more detailed annotations in which different cell types are either annotated by a point/dotting (for cell detection and classification) or by marking the boundary of nuclei in a free-hand manner if cell segmentation is necessary as part of the downstream analysis. Figure S3 shows both point and free-hand (polygon) annotations of different cells. Various features are extracted from cells and their environment for downstream diagnostic or prognostic purposes. These features include morphology, cell counts, cell-to-cell ratios, cell graphs, and cellular colocalisation.

| 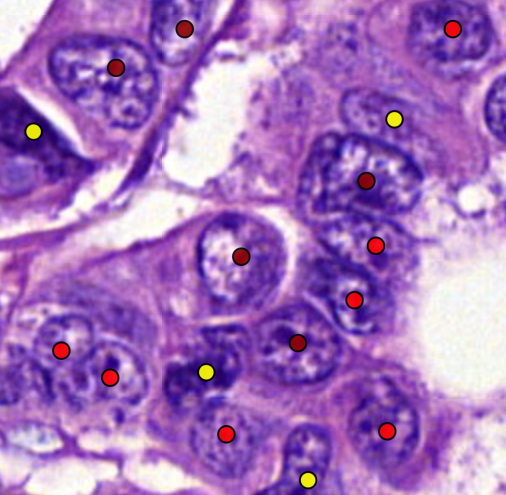 | 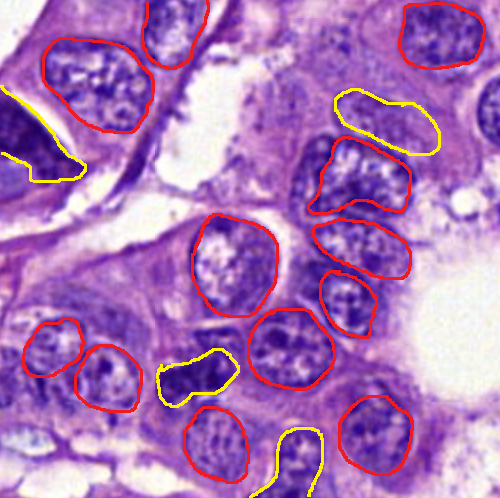 |
| --- | --- |
| A | B |

**Figure S3.** A) Point-based cell annotation, B) Free-hand polygon-based cell annotation. Different colours indicate different cell classes.

### **S2.2.4 Descriptive and multi-modal annotations**

Annotations in the form of textual description such as a pathology report or text annotation of an area of interest on the WSI can also be recorded. For example, BCa pathology report provides clinically relevant information including diagnosis, invasive tumour size, lymph node status and potential targets for treatment (e.g., hormone receptor status). A natural language processing system can be used to automate the process of extracting useful information from the descriptive annotations but as the descriptions may vary from laboratory to laboratory, it might be challenging to achieve high accuracy [*52].

In addition, with a growing interest in the integration of histopathology with genomics and transcriptomics, associated data can be collected from other sources to augment imaging information to advance patient care [*39, *53]. Genomic, epigenomic, transcriptomics, proteomic and imaging data can be obtained from different sources such as The Cancer Genome Atlas (TCGA) and Clinical Proteomic Tumour Analysis Consortium (CPTAC).

## **S2.3 Annotation constructs**

Depending on the types of annotations different annotation construct/tools (Figure S4) could be used to mark the structures e.g., regions, cells.

**Bounding box:** Bounding boxes are axis-aligned rectangles which can be used for enclosing ROIs, or for enclosing other constructs such as polygons, lines, and points. Bounding boxes may be used for exhaustive annotations as it is almost impossible to annotate the WSI completely. A ‘consensus box’ can be drawn to allow multiple pathologists to annotate the same ROI. This box can be analysed to assess concordance between experts when assessing key features within a WSI. For example, if a box is defined for region-level annotation, then the annotators can concentrate fully on this area while annotating. This also makes it easier for automation scripts to extract relevant annotation for ML purpose. Boxes can be further categorised, such as a region-box, cell-box, individual-annotation-box, consensus-annotation-box, etc.

**Point/circle/dot:** A point annotation is used to annotate cells or regions. This type of annotation is quicker but less precise as it will only provide a nominal centre. If a more detailed annotation is required, such as nuclei or cell boundary, then a polygon or free-hand drawing is recommended.

**Polygon:** A polygon can be used to provide additional precision to a region/cell annotation.

**Line:** A straight line can be drawn to segregate two neighbouring structures, join two structures of interest, mark axis (e.g., major-axis or minor-axis) of a region/gland or cell, measure the diameter of an object or measure distance between two objects.

**Text:** Textual annotation can be used to describe regions and cells in the surrounding context. This annotation acts as a meta-annotation for other annotations.


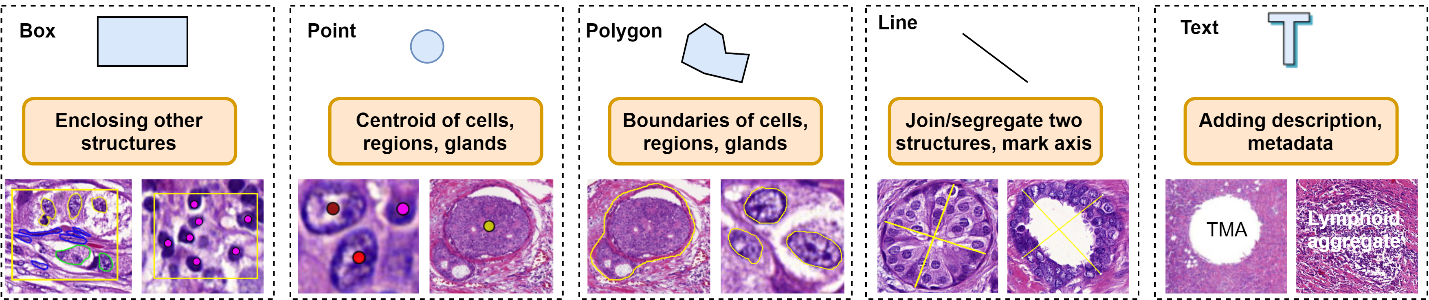


**Figure S4.** Different tools that can be used for annotations.

## **S2.4 Degree of annotation**


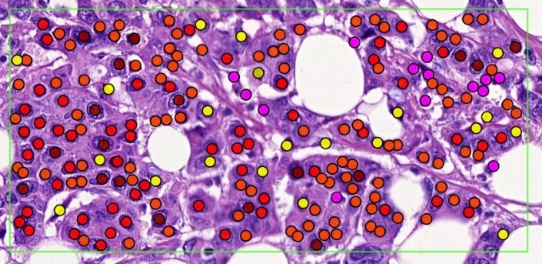

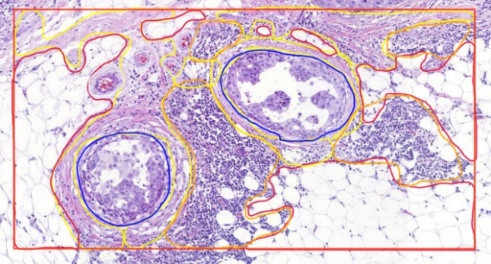

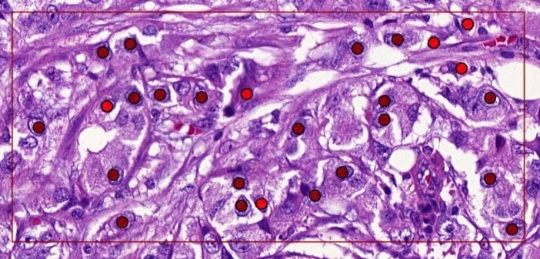

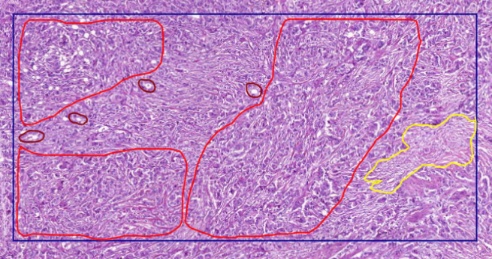


A

B

C

D

**Figure S5.** Exhaustive (A, B) vs non-exhaustive (C, D) annotations. A & B) Exhaustive cell-level and region-level annotations where all the cells/regions in the bounding box are annotated. C & D) Non-exhaustive cell-level and region-level annotations where some of the cells/regions in the bounding box are not annotated.

## **S2.5 Phases of annotation**


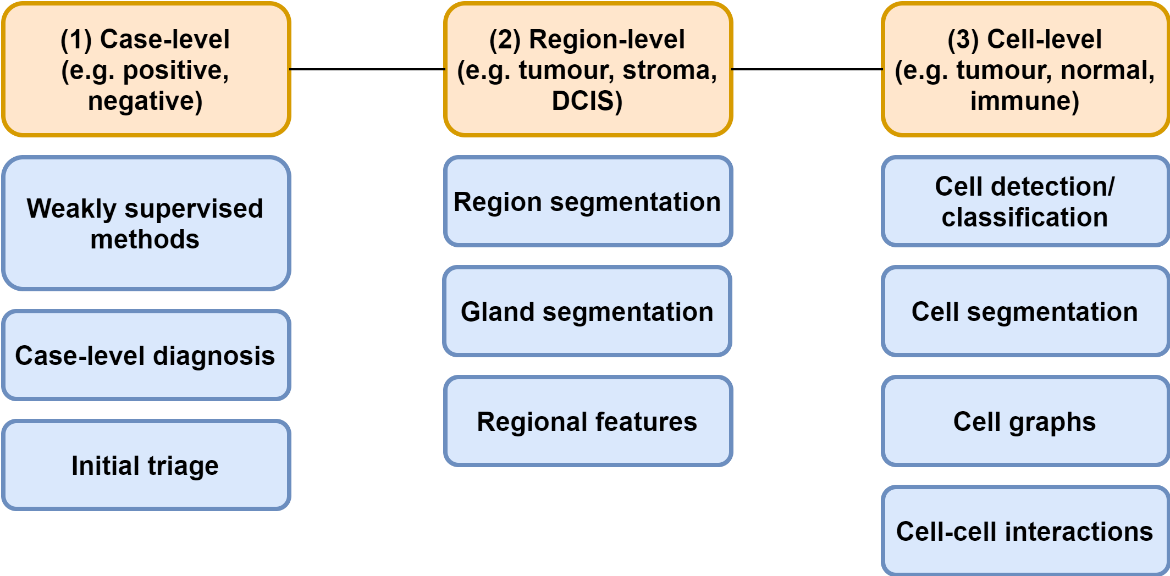


**Figure S6.** Proposed phases of annotations with their corresponding machine learning usage. ‘Initial triage’ means using a trained ML model for classifying most of the negative cases with high confidence so that pathologists can give more time to the difficult cases.

## **S2.6 Interactive and active annotations**

Interactive annotations can expedite the annotation process where the output of an annotation model are reviewed by an expert to provide feedback for the improving the performance of the model. Similarly, in an active learning-based framework the model improves its performance in an iterative manner by asks the expert for samples from an unannotated dataset.

For the segmentation task (requiring dense annotation), interactive approaches bring several advantages over fully automated approaches: 1) the supervisory signal acts as a prior to the interactive model, and leads to better performance; 2) interactive models are less sensitive to domain shift and are more generalisable [16]; and 3) interactive models give the flexibility to the user to choose arbitrary instances of objects present in the visual field – for instance, selecting and focusing on only some of the nuclei to be segmented, instead of segmenting all of them using an automatic method. Figure S7 shows how an interactive segmentation tool (NuClick [16]) can be used to generate nuclei and gland masks from point and squiggle annotations.

Similarly, active learning-based annotations can also help to reduce the need for manual annotations and speed up the of annotation process [*54]. In this setting, an ML algorithm (for example, a region segmentation model) utilises a limited annotation set for training. It then produces annotations as output which are then confirmed or modified by a pathologist. The augmented annotations are reused for training the model and the process is repeated until a desired level of accuracy is achieved.

Mitosis Normal epithelial Stromal cell TILs

Tumour NP1 Tumour NP2 Tumour NP3

A

B


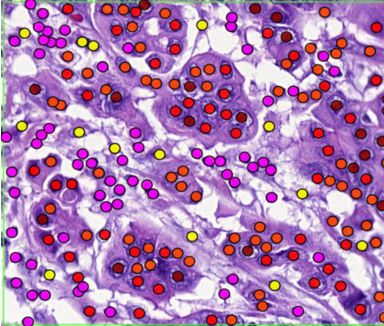

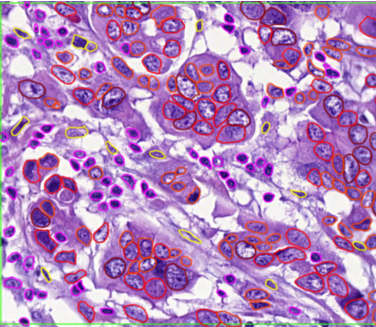


**Figure S7.** NuClick interactive segmentation. A) Point annotation for H&E cells, B) NuClick generated segmentation masks.

## **S2.7 Quality of tissue sectioning, staining and scanning**

### **S2.7.1 Tissue sectioning quality**

For accurate and reliable quantitative analysis, enough tissue should be present on the slide. To avoid any false signal during ML based analysis, the section should be checked for any damage such as tears, folds, creases, and knife marks. The presence of artefacts such as blurriness, pen-markings, and tissue folding could pose major challenges to the downstream analyses. They are normally unintentionally introduced during both routine slide preparation and digitisation. These kinds of artefacts can be manually excluded during annotation and review. However, manual review is time consuming, laborious, and subject to intra- and inter-reader variability therefore an automated QC of images is required. Slides which consist of large artefacts regions should be excluded directly before further annotation and computational analysis.

### **S2.7.2 Staining quality**

Proper SOPs should be followed for staining the slides [*55,*56]. Staining issues could hamper initial analytical tasks such as nuclei detection and segmentation and any further downstream analyses relying on these steps. Ideally, we would expect an ML pipeline to work on cases deemed reportable by pathologists but this is not always the case because of the complex nature of ML models. Staining intensity should be harmonised by stain deconvolution steps to avoid problems of differences in performance by different scanners and laboratories [*57].

### **S2.7.3 Scanning quality**

Though each digital whole slide scanner may have its own requirements and workflow, certain standards should be followed while scanning glass slides [*58]. The images should be checked for scanner resolution, out-of-focus, scan line and background artefacts.

## **S2.8 Image quality control**

ImageQC workflow includes five steps: i) Pen markings detection, ii) Coverslip edge detection, iii) Tissue detection, iv) Blurry region identification, and v) Morphological operations (Figure S8). Briefly, each whole slide image goes through pen-markings and coverslip edge detections to remove shadow areas and pen markings, followed by the tissue region detection in the non-pen-markings regions. Then blur detection method [*59] is used to detect the blurriness in the detected tissue region. The blur detection method generates a blurred version of the input image and then estimates blurriness based on the variation between neighbouring pixels in the input versus blurred images. Based on empirical evidence, slides with more than 60% blurry regions will be excluded for further analysis and returned for rescanning. After getting the artefact free regions using the first four steps, we then perform several morphological operations to generate a single tissue mask which is ready for further annotation and computational analysis. These operations remove small, disconnected foreground objects, and fill small background holes in the mask.


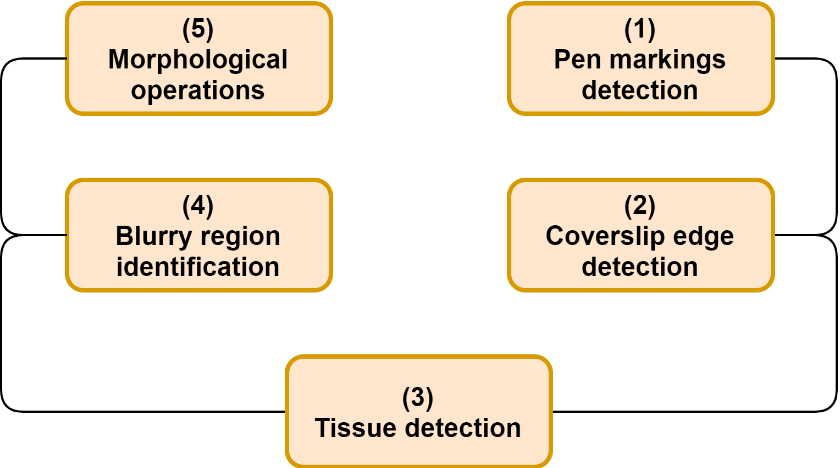


**Figure S8.** Workflow of ImageQC

## **S2.9 Measuring inter-annotator agreement**

When measuring inter-annotator agreement, the exhaustiveness may not often reach 100% because for some regions it may be impossible to discern precise start and end points or are subjective in whether they comply precisely with the definition. The annotators may sometime omit an object because of its lack of relevance to the annotation protocol. Likewise, where the regions are annotated by multiple annotators, exhaustiveness will be calculated for regions on which all the annotators agree.

To measure such inter-annotator agreement, the Jaccard similarity index (JSI) (eq. 1) which calculates the ratio of the intersection of the annotated areas to the union of the annotated areas can be used. In eq. 1, if A and B represent the regions annotated by two different annotators, $|A\cap B|$ is the number of elements (pixels) where annotations of two annotators intersect and $|A\cup B|$ would be the union of both A and B annotations. The value of the JSI range between 0 (no agreement at all) and 1 (100% agreement).

$JSI(A, B)=\frac{|A\cap B|}{|A\cup B|}$ eq. 1

Other similar metrics such as Dice Similarity index (DSI) also called Dice Index or Dice coefficient (eq. 2) could also be used instead of JSI. DSI also ranges between 0 and 1.

$DSI(A, B)=\frac{2|A\cap B|}{|A\cup B|}$ eq. 2

To measure inter-rater reliability of cell annotations by two annotators Cohen’s Kappa can be used (eq. 3) where $p_{o}$ and $p_{e}$are the observed and hypothetical probability of chance agreement between two annotators, respectively.

$Cohen kappa=\frac{p_{o}-p_{e}}{1-p_{e}}$ eq. 3

In case of more than two annotators Fleiss’ Kappa (eq. 4) can be used where $\bar{P}$ and $\bar{P}_{e}$ are the mean observed and hypothetical probability of chance agreement between multiple annotators, respectively. The main difference of Fleiss Kappa from the Cohen Kappa is in terms of taking more than two annotators into account by computing agreement in rater-rater pairs relative to all possible pairs and taking the mean.

$Fleiss kappa=\frac{\bar{P}-\bar{P}_{e}}{1-\bar{P}_{e}}$ eq. 4

## **S2.10 Pathologists’ review of annotation**

We recommend a regular review of the annotations by pathologists so that any major issues in the annotation process can be identified and rectified in a timely manner. The review process can also cover any issues brought up during the QC. Relatively minor issues can include improper use of styles, annotation constructs etc., and these may be resolved by the individual pathologist to whom those annotations were assigned. Other important issues such as disagreement on features should be submitted according to the prioritisation list, starting with the highest priority regions or cells. These issues need to be discussed for resolution at the periodic review meetings. A selected set of cases might also be used to measure intra-annotator reproducibility.

# **Section S3. Results (Applied example)**

## **S3.1 Annotation data dictionary**

Figure S9 shows the different types of bounding boxes and describes their purpose. Table S2 further describes each bounding box, for example, Box_All_Cell_20x is required for cell-level consensus annotations by multiple pathologists. Tables S3 and S4 describe each region and cell types, respectively that are required to be annotated. Finally, Table S5 lists all the styles (name, style name, annotation shape, line colour, and the Red Green Blue (RGB) values for the line colours) to differentiate them visually more easily as well as for ML purposes.


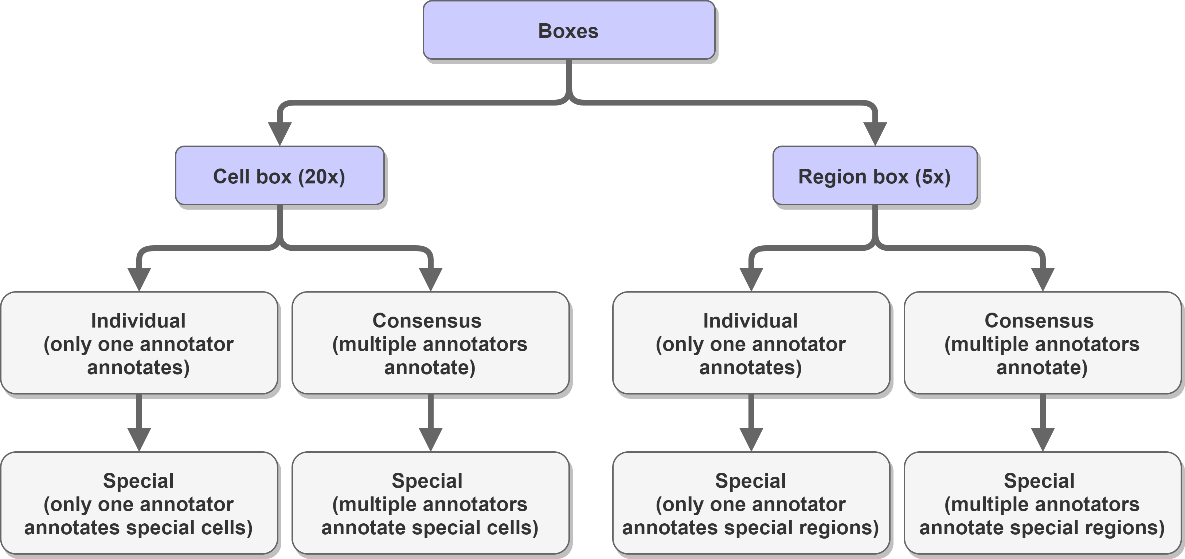


**Figure S9.** Sample tree diagram of different bounding boxes from PathLAKE annotation data dictionary

**Table S2.** Description of bounding boxes used for region annotations in PathLAKE annotation data dictionary.

| **Term** | **Definition** |
| --- | --- |
| Cell Box Individual  (Box_Cell_20x) | A box in which each cell is annotated by one annotator only.  A box drawn at 20x magnification to cover a visual field. |
| Region Box Individual  (Box_Region_5x) | A box in which each region is annotated by one annotator only.  A box drawn at 5x magnification to cover a visual field. |
| Cell Box Consensus  (Box_All_Cell_20x) | A box in which each cell is annotated by multiple annotators.  A box drawn at 20x magnification to cover a visual field. |
| Region Box Consensus  (Box_All_Region_5x) | A box in which each region is annotated by multiple annotators.  A box drawn at 5x magnification to cover a visual field. |
| Cell Box Individual Special  (Box_Special_20x) | A box in which each cell of special type (e.g., mitosis) is annotated by one annotator only.  A box of an appropriate size drawn at 20x magnification. Every cell of the special type inside this box is annotated by a single annotator only. It is used for mitosis, normal epithelial, and tumour cells. |
| Region Box Individual Special  (Box_Special_5x) | A box in which each region of special type (e.g., DCIS) is annotated by one annotator only.  A box of an appropriate size drawn at 5x magnification. Every region of the special type inside this box is annotated by a single annotator. It is used for DCIS, vascular channels, lymphoid follicles, normal TDLUs, etc. |
| Cell Box Consensus Special  (Box_ALL_Special_20x) | A box in which each cell of special type (e.g., mitosis) is annotated by multiple annotators.  A box of an appropriate size drawn at 20x magnification. Every cell of the special type inside this box is annotated by multiple annotators. It is used for mitosis, normal epithelial, and tumour cells. |
| Region Box Consensus Special  (Box_ALL_Special_5x) | A box in which each and every region of special type (e.g., DCIS) is annotated by multiple annotators.  A box of an appropriate size drawn at 5x magnification. Every region of the special type inside this box is annotated by multiple annotators. It is used for DCIS, vascular channels, lymphoid follicles, normal TDLUs, etc. |

**Table S3.** Description of region annotations for breast H&E from PathLAKE annotation data dictionary

| **Region name** | **Description** | **Example** |
| --- | --- | --- |
| **Normal** | | |
| Normal TDLUs | Normal terminal ductal lobular units (TDLUs) | 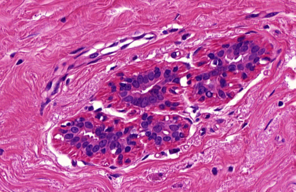 |
| Normal skeletal muscles | Normal skeletal muscles | 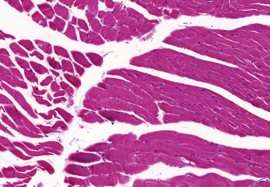 |
| Normal nerves | Normal Nerve bundles | 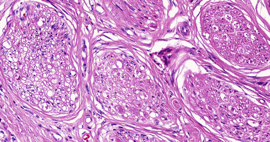 |
| Normal vascular channels | Normal vascular channels (blood vessels or lymphatic channels) | 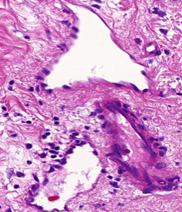 |
| **Benign/non-malignant lesions** | |  |
| Benign papilloma | Benign papilloma are benign tumours that grow from epithelial tissue and form outward finger-like fronds with fibrovascular cores. | 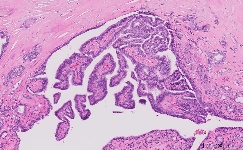 |
| Fibroadenoma | Fibroadenomas are made up of overgrowth of both glandular tissue and stroma tissue. | 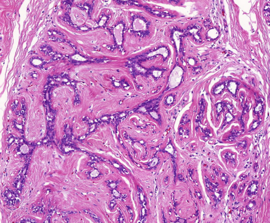 |
| Fibrocystic change | Fibrocystic change. Dilated cysts along with areas of fibrosis. | 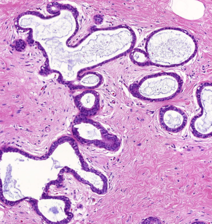 |
| Usual ductal hyperplasia | Hyperplasia is an overgrowth of the cells that line the breast ducts. Cells are not neoplastic. | 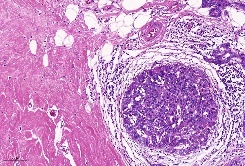 |
| Benign others | Any other benign lesion that can be present in a given slide such as radial scar, microglandular adenosis, lactational changes, apocrine metaplasia, etc. | 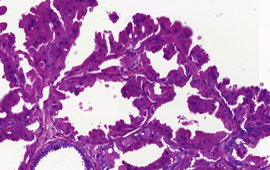 |
| **DCIS/LCIS** | |  |
| DCIS | Ductal carcinoma in situ (DCIS) is a non-invasive form of BC which is defined as neoplastic epithelial cells proliferating within the mammary ducts, which have not breached the basement membrane nor invaded surrounding tissues. | 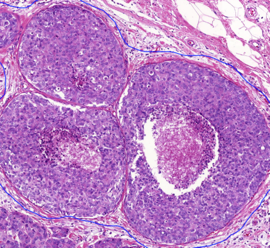 |
| Lobular neoplasia (LN) | Lobular neoplasia is a condition in which atypical cells are found in the terminal lobules of the breast. This category includes atypical lobular hyperplasia and lobular carcinoma in situ (LCIS). | 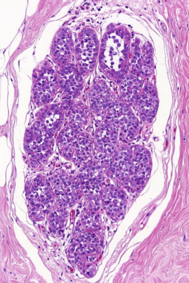 |
| **Stroma** | |  |
| Stroma fibroblastic | Fibroblasts are connective tissue which produces collagen and other fibres. This type of stroma is found around non-malignant lesions. | 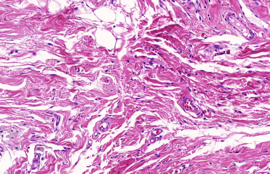 |
| Stroma tumour associated | Complex Stroma (tumour associated) mainly consists of the basement membrane, fibroblasts, myofibroblasts extracellular matrix, immune cells, and vasculature. This type of stroma is associated with malignant lesions. | 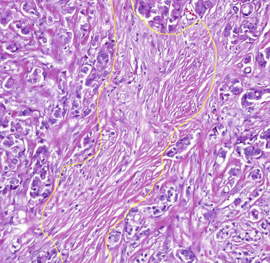 |
| Stroma fibrofatty | Stroma formed of adipocytes (large clear cells with peripherally placed compressed nuclei) intersected with thin or thick fibrous tissue stroma. | 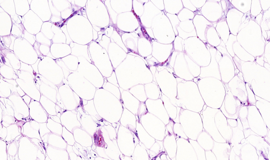 |
| **Tumour** | |  |
| Tumour tubules acini | Tubule/Acinar formation. The tumour exhibits tubule formation like normal breast. | 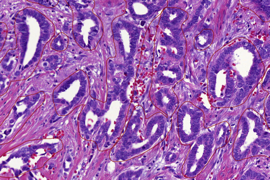 |
| Tumour non tubular | Tumour (Non-tubular) Sheets/cords, nests, single cells | 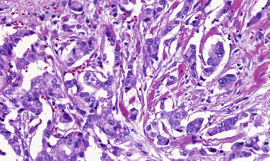 |
| Tumour mucinous | It is a rare form of invasive ductal carcinoma made up of malignant epithelial cells that float in pools of mucin. | 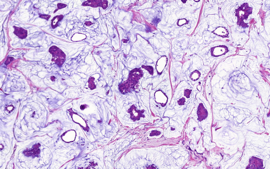 |
| Tumour invasive mixed | Invasive tumour mixed pattern. This includes more than one invasive tumour pattern in a single lesion, for example ductal and lobular in a single image. | 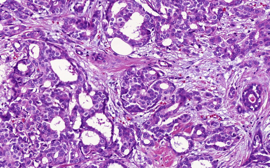 |
| **Other** | |  |
| Lymphoid follicle | Aggregates of lymphocytic and histiocytic infiltrate plasma cells. Often shows germinal cells formation. | 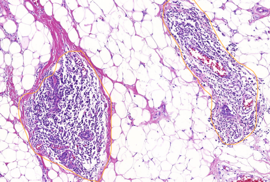 |
| Lympho-vascular invasion | It is malignant cells within blood vessels and/or lymphatics. | 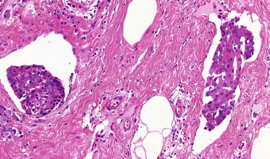 |
| Perineural invasion | Perineural invasion (PNI) is described when cancer cells encroach along nerves. | 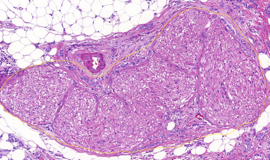 |
| Exclusion | Areas of exclusion include folding, Tissue Micro Array site, background, ink, out of focus regions and air bubbles) | 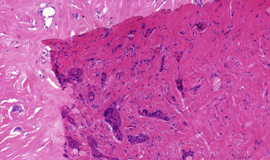 |
| Extra features | Extra-features such as biopsy site changes, calcifications, necrosis, and haemorrhage | 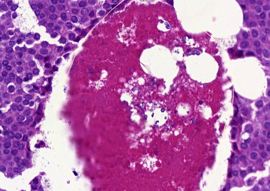 |

**Table S4.** Description of cell annotations for breast H&E from PathLAKE annotation data dictionary

| Cell name | Description | Example |
| --- | --- | --- |
| Mitosis | Mitosis either typical (prophase, metaphase, anaphase, and telophase) or atypical (anaphase bridge, multipoles, and asymmetrical shape) | 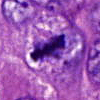 |
| Normal epithelial/  myoepithelial | Normal epithelial cells are the innermost layer of bilayered ductolobular system and  Myoepithelium: outermost layer resting on a basement membrane | 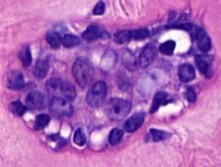 |
| Epithelial NP1 (nuclear pleomorphism 1) | Tumour cells which are similar in size to normal epithelial cells. | 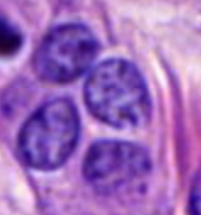 |
| Epithelial NP2 (nuclear pleomorphism 2) | Tumour cells with moderate variability in size and shape. They are larger in size with open vesicular nuclei and visible nucleoli. | 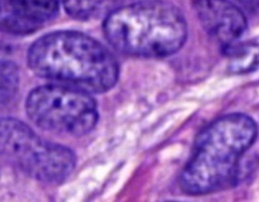 |
| Epithelial NP3 (nuclear pleomorphism 3) | Tumour cells with high variability in size and shape. | 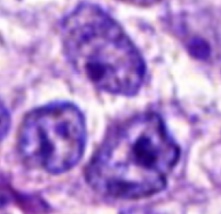 |

**Table S5.** Annotation styles for regions (Breast H&E) which could be used to generate a style file (such as in JSON format) to be used with an annotation software. (From PathLAKE annotation data dictionary)

| **Type** | **Style name** | **Annotation shape** | **Line Colour** | **RGB/HEX Values** |
| --- | --- | --- | --- | --- |
| Normal TDLUs | HE_Region_normal_TDLUs | Polygon | Green | 0,128,0  #008000 |
| Normal skeletal muscles | HE_Region_normal_skeletal_ms | Polygon | Light Green | 144,238,144  #90EE90 |
| Normal nerves | HE_Region_normal_nerves | Polygon | Dark Sea Green | 143,188,143  #8FBC8F |
| Normal vascular channels | HE_Region_normal_vascular_ch | Polygon | Lavender | 230,230,250  #E6E6FA |
| Papillomas | HE_Region_benign_papillomas | Polygon | Deep Pink | 255,20,147  #FF1493 |
| Fibroadenoma | HE_Region_benign_fibroadenoma | Polygon | Aqua | 0,255,255  #00FFFF |
| Fibrocystic change | HE_Region_benign_fibrocystic_change | Polygon | Lime | 0,255,0  #00FF00 |
| Usual ductal hyperplasia | HE_Region_benign_hyperplasia | Polygon | Coral | 255,127,80  #FF7F50 |
| Benign others | HE_Region_benign_others | Polygon | Cadet Blue | 95,158,160  #5F9EA0 |
| DCIS | HE_Region_DCIS | Polygon | Blue | 0,0,255  #0000FF |
| Lobular neoplasia | HE_Region_lobular_neoplasia | Polygon | Light Sky Blue | 135,206,250  #87CEFA |
| Stroma fibroblastic | HE_Region_stroma_fibroblastic | Polygon | Light Yellow | 255,255,102  #FFFF66 |
| Stroma tumour associated | HE_Region_stroma_tumor_associated | Polygon | Yellow | 255,255,0  #FFFF00 |
| Stroma fibrofatty | HE_Region_stroma_fibrofatty | Polygon | Crimson | 220,20,60  #DC143C |
| Tumour mucinous | HE_Region_tumor_mucinous | Polygon | Orange Red | 255,69,0  #FF4500 |
| Tumour invasive mixed | HE_Region_ tumor_invasive _mixed | Polygon | Light Red | 224,102,102  #E06666 |
| Tumour tubules acini | HE_Region_tumor_tubules_acini | Polygon | Dark Red | 139,0,0  #8B0000 |
| Tumour non tubular | HE_Region_tumor_non_tubular | Polygon | Red | 255,0,0  #FF0000 |
| Lymphoid | HE_Region_lymphoid | Polygon | Orange | 255,165,0  #FFA500 |
| Lympho-vascular invasion | HE_Region_LVI | Polygon | Dark Magenta | 139,0,139  #8B008B |
| Perineural invasion | HE_Region_PNI | Polygon | Gold | 255,215,0  #FFD700 |
| Exclusion | HE_Region_exclusion | Polygon | Black | 0,0,0  #000000 |
| Extra features | HE_Region_extra_features | Polygon | Cream White | 255,253,208  #FFFDD0 |

## **S3.2 Annotation software**

Due to the distributive nature of the project the locations and time constraints of the pathologists, an open-source online annotation software (Digital Slide Archive (DSA) [28]) was customised and extended and adapted into the WArwick Slide Archive and collaBoration Interface (WASABI). As the current version of the DSA software lacked some features such as workload distribution, an annotation download interface, annotation status (as a leader board), grouping of annotations a frontend gateway was built for this purpose. Annotations were saved in JSON format following a well-defined file naming convention. To add further features such as ML results visualisation, side-by-side image comparison, integration of interactive annotations, and to incorporate other features requested by pathologists and ML experts, an advanced version (WASABI+) is currently under development.

## **S3.3 Annotation levels**

Annotations were conducted at three levels (case-level, region-level, and cell-level). Table S6 lists the details of annotations made at different levels. Note that the case-level annotations differ for the different stains because of some cases dropped during QC. Figure S2 shows some sample region-level annotations where different types of regions are annotated in different colours. Similarly, Figure S3 shows both point and free-hand polygon annotations of different types of cells.

Seventy cases were used for consensus as well as individual annotations whereas the rest were used for annotations by individual annotators only. The consensus annotations were completed by two annotators. Tables S7-S12 list details of the numbers and areas of region-level and counts of cell-level annotations for both H&E and IHC stained slides. Region-level annotations were done at 5$\times$ magnification whereas cell-level annotations were done at 20$\times$ magnification. For each case two bounding boxes were drawn for regions and two bounding boxes for cells.

Annotations were completed in different batches of seven sets whereas each set comprise 70-80 cases and each has 3 images (H&E, PR and Ki67) to allow modular annotation and to analyse and compare them properly.

**Table S6.** Examples of number of annotations at different levels. Case-level (grades), Region-level (different regions were annotated using a polygon. Number of region annotation as well as the area in mm^2^ is listed). Cell-level (different types of cells were annotated using a dot/point).

| **Case-level** | | | **Region-level** | | | | | | **Cell-level** | | |
| --- | --- | --- | --- | --- | --- | --- | --- | --- | --- | --- | --- |
| **H&E** | **PR** | **Ki67** | **H&E** | | **PR** | | **Ki67** | | **H&E** | **PR** | **Ki67** |
| 1226 | 1143 | 1121 | **Count** | **Area**  **(mm^2^)** | **Count** | **Area**  **(mm^2^)** | **Count** | **Area**  **(mm^2^)** | 249834 | 204317 | 57388 |
|  |  |  | 79494 | 2215.37 | 32809 | 716.80 | 27490 | 864.81 |  |  |  |


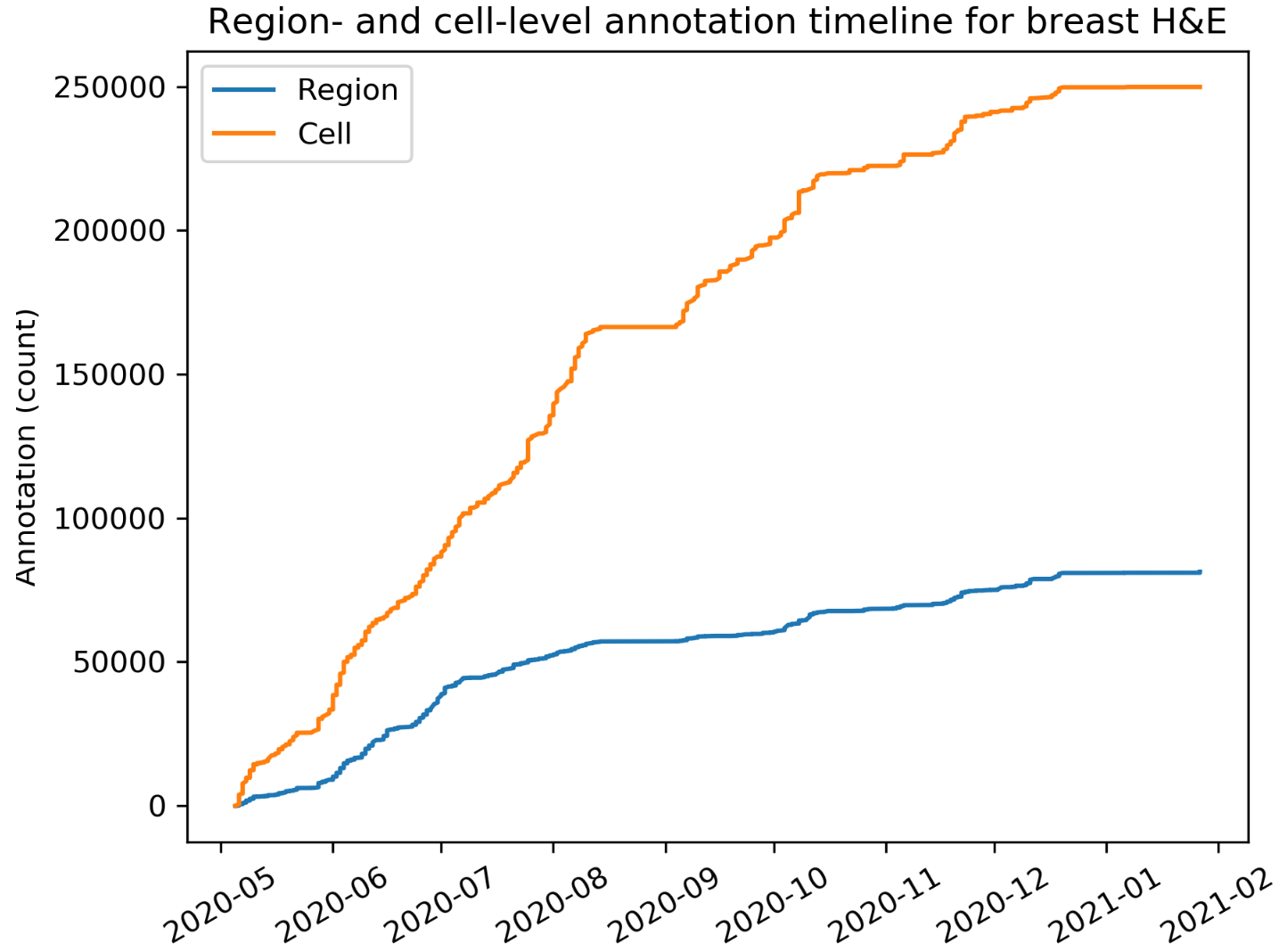


A

C


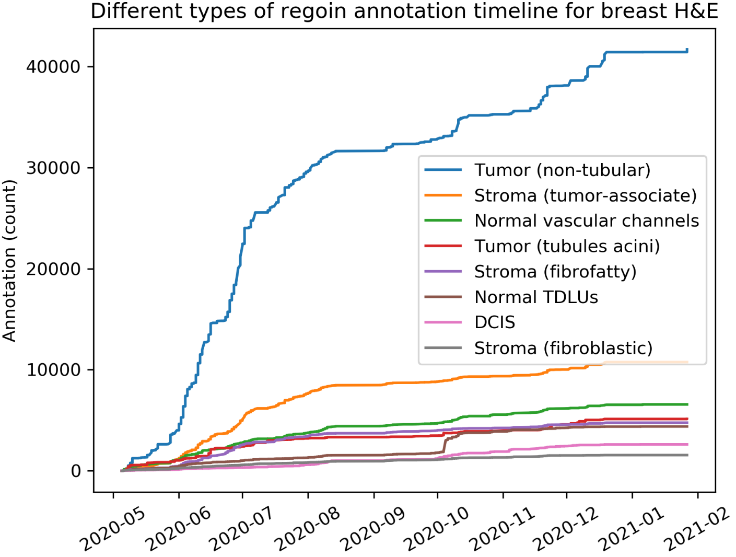

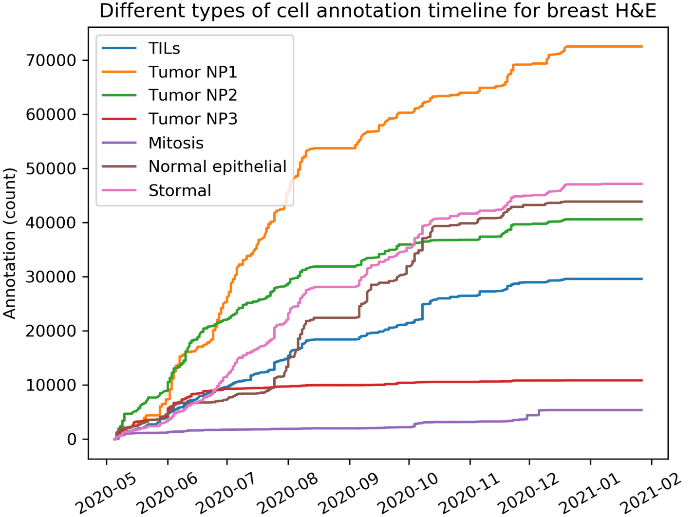


B

**Figure S10.** A) Timeline of overall region- and cell-level annotations for breast H&E showing increase in the counts of annotations over time; B) Timeline of different types of breast H&E regions (top 8); C) Timeline of different types of breast H&E cells. NP (nuclear pleomorphism)

**Table S7.** Region-level annotations for breast cancer H&E WSIs

| Region | Count | Area (mm^2^) |
| --- | --- | --- |
| Fibroadenoma | 2 | 1.19 |
| Fibrocystic change | 34 | 17.32 |
| Usual ductal hyperplasia | 100 | 9.84 |
| Benign others | 36 | 11.34 |
| Papillomas | 8 | 2.76 |
| DCIS | 2714 | 395.21 |
| Exclusion | 1,229 | 43.26 |
| Extra features | 638 | 43.05 |
| Lobular neoplasia | 421 | 16.92 |
| Lympho-vascular invasion (LVI) | 303 | 20.82 |
| Lymphoid | 443 | 36.54 |
| Normal nerves | 275 | 9.13 |
| Normal skeletal muscle | 212 | 68.60 |
| Normal TDLUs | 4,383 | 142.77 |
| Normal vascular channels | 7,058 | 30.38 |
| Perineural invasion | 20 | 1.22 |
| Stroma fibroblastic | 1,628 | 151.51 |
| Stroma fibrofatty | 4,851 | 247.54 |
| Stroma tumour associated | 11,050 | 209.11 |
| Tumour invasive mixed | 124 | 9.52 |
| Tumour mucinous | 305 | 360.66 |
| Tumour non tubular | 42,750 | 359.52 |
| Tumour tubules acini | 5,293 | 27.04 |
| Total | **79,494** | **2,215.37** |

**Table S8.** Region-level annotations for breast cancer PR WSIs

| Region | Count | Area (mm^2^) |
| --- | --- | --- |
| DCIS | 1,740 | 290.56 |
| Exclusion | 589 | 43.68 |
| Lymphoid Follicles | 381 | 50.56 |
| Normal | 3,687 | 113.80 |
| Tumour | 26,407 | 218.16 |
| Total | **32,804** | **716.76** |

**Table S9.** Region-level annotations for breast cancer Ki67 WSIs

| Region | Count | Area (mm^2^) |
| --- | --- | --- |
| DCIS | 1,586 | 330.26 |
| Exclusion | 589 | 59.20 |
| Ki67 hotspot* | 74 | 18.26 |
| Lymphoid Follicles | 388 | 58.90 |
| Normal | 3343 | 105.48 |
| Tumour | 21,510 | 292.72 |
| Total | **27,490** | **864.81** |

*Not annotated in all images. Only few examples annotated to compare between the performance of ML algorithm and pathologists.

**Table S10.** Cell-level annotations for breast cancer H&E WSIs

| Cell type | Count |
| --- | --- |
| Mitosis | 5,354 |
| Normal epithelial | 43,852 |
| Stroma | 47,123 |
| Tumour infiltrating lymphocytes (TILs) | 29,581 |
| Tumour Nuclear pleomorphism 1 (NP1) | 72,492 |
| Tumour NP2 | 40,590 |
| Tumour NP3 | 10,842 |
| Total | **249,834** |

**Table S11.** Cell-level annotations for breast cancer PR WSIs

| Tumour cell nuclear staining | Count |
| --- | --- |
| Negative | 56,210 |
| Positive moderate intensity | 57,140 |
| Positive strong intensity | 46,133 |
| Positive weak intensity | 44,834 |
| Total | **204,317** |

**Table S12.** Cell-level annotations for breast cancer Ki67 WSIs

| Cell type | Count |
| --- | --- |
| Negative non-tumour | 19,223 |
| Negative tumour | 26,308 |
| Positive* non-tumour | 2,424 |
| Positive tumour | 9,433 |
| Total | **57,388** |

*Any staining pattern and intensity were considered as positive in Ki67.

## **S3.4 Annotation constructs**

Three main annotation constructs: rectangle, point, and polygon, were used to annotate different structures as regions of interest, cell points, and region boundaries. In total 10,731 rectangles, 509,591 points, and 194,717 polygons were used for annotations (including both H&E and IHC). Further detail of the use of constructs is provided in Table S13.

**Table S13.** Usage of annotation constructs (rectangles) for cell and region annotation

| Construct | Count |
| --- | --- |
| Box_All_Cell_20x | 281 |
| Box_All_Region_5x | 80 |
| Box_ALL_Special_20x | 288 |
| Box_ALL_Special_5x | 67 |
| Box_Cell_20x | 1,792 |
| Box_Region_5x | 1,485 |
| Box_Special_20x | 3,518 |
| Box_Special_5x | 3,220 |
| Total | **10,731** |

## **S3.5 Degree of annotation**

Annotations were completed in an exhaustive manner: all the different types of regions and cells defined in the data dictionary within a bounding box were annotated by the pathologists. To quantify this, the proposed exhaustiveness metric was used to identify non-exhaustive annotations. The diversity in Figure S11 represents how many different types of regions were annotated in a region box. The overall exhaustiveness of the annotations is high with most of the boxes having higher than 80% exhaustiveness for individual boxes (represented by red dots) and above 60% exhaustiveness for consensus boxes (represented by blue dots). Similarly, most of the boxes contain between 3 to 6 types of regions. Figure S4 shows examples of exhaustive region and cell annotations.


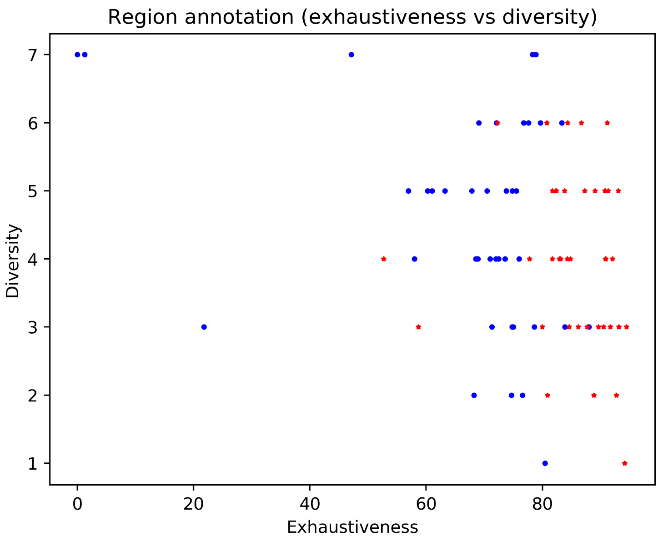


**Figure S11.** Exhaustiveness vs diversity of the region box annotation where each dot represents a region box. Blue dots represent consensus boxes whereas red dots represent individual boxes.

## **S3.6 Phases of annotation**

To familiarise the multi-disciplinary team with the definitions of different structures and identify any potential annotation issues, two workshops were held where pathologists and ML experts participated. For this purpose, a small set of representative cases (those showing stain variations, tissue size, stain types, and structural diversity) were assigned for a trial annotation to pathologists in groups. This pilot phase not only helped in identifying useful structures but also helped in annotation workload estimation. Once the pathologist team was acquainted with the protocol and the tools, the annotations proceeded in a phased manner, starting with case-level labels. Diagnostic/prognostic records were checked and were compiled into a single structured form as clinical data is often unstructured and maybe distributed across disparate medical information systems. Relevant cells and regions were then annotated to help with detailed supervision of the ML methods.

## **S3.7 Interactive/active annotations**

As annotation of cells/nuclei boundaries is very time consuming, we used NuClick [16], a unified interactive segmentation framework for histopathological images to generate such nuclei segmentation masks. It requires only one point for delineating nuclei and cells and a squiggle for outlining glands or larger areas. Providing a dot inside a nucleus and cell or drawing a squiggle inside a gland is fast, easy, and combined with good performance of the model, makes NuClick user-friendly. Also, it allows for converting existing low-level annotations (points) into dense annotations (segmentation masks). Figure S7 illustrates example outputs of NuClick annotation in response to low-level guidance of annotator for nuclei boundary segmentation. Using this framework, we generated a total of 124,624 H&E and 109,862 PR cell boundaries for the BraCe project.

Mitosis Normal epithelial Stromal cell TILs

Tumour NP1 Tumour NP2 Tumour NP3

A

B


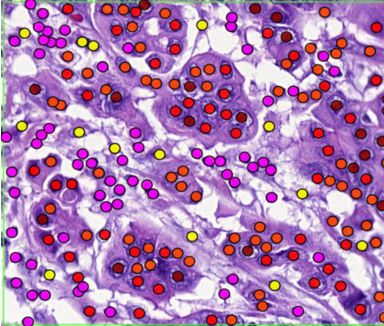

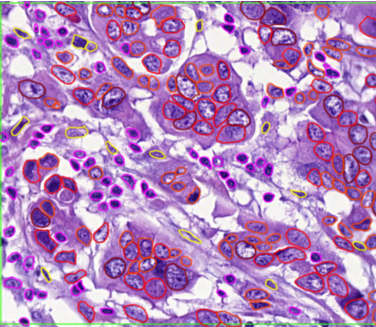


**Figure S7^** NuClick interactive segmentation. A) Point annotation for H&E cells, B) NuClick generated segmentation masks. (^Figure replicated for ease of reference)

## **S3.8 Workload distribution**

Based on the pilot phase and some initial annotations, the workload distribution was estimated. The pathologists were put into three teams with a mix of experience in each team, and the slides for annotation were split equally amongst the teams. As the project progressed, some annotators completed their work more quickly than other staff of different time pressures and clinical commitments. Groups of two pathologists with a mix of experiences were formed so that consensus annotations could be done.

## **S3.9 Quality review**

### **S3.9.1 Image Quality analysis**

The results of ImageQC pipeline on WSIs with pen-marking, coverslip edges and blurriness are shown in Figure S12. It can be observed that the blue, green, and dark green pen-markings, shadow region on the coverslip edges (Figure S12 A and B) have been adequately removed during the workflow. Similarly, Figure S12 C shows the detection of pen-marking and blurriness in two other WSIs.


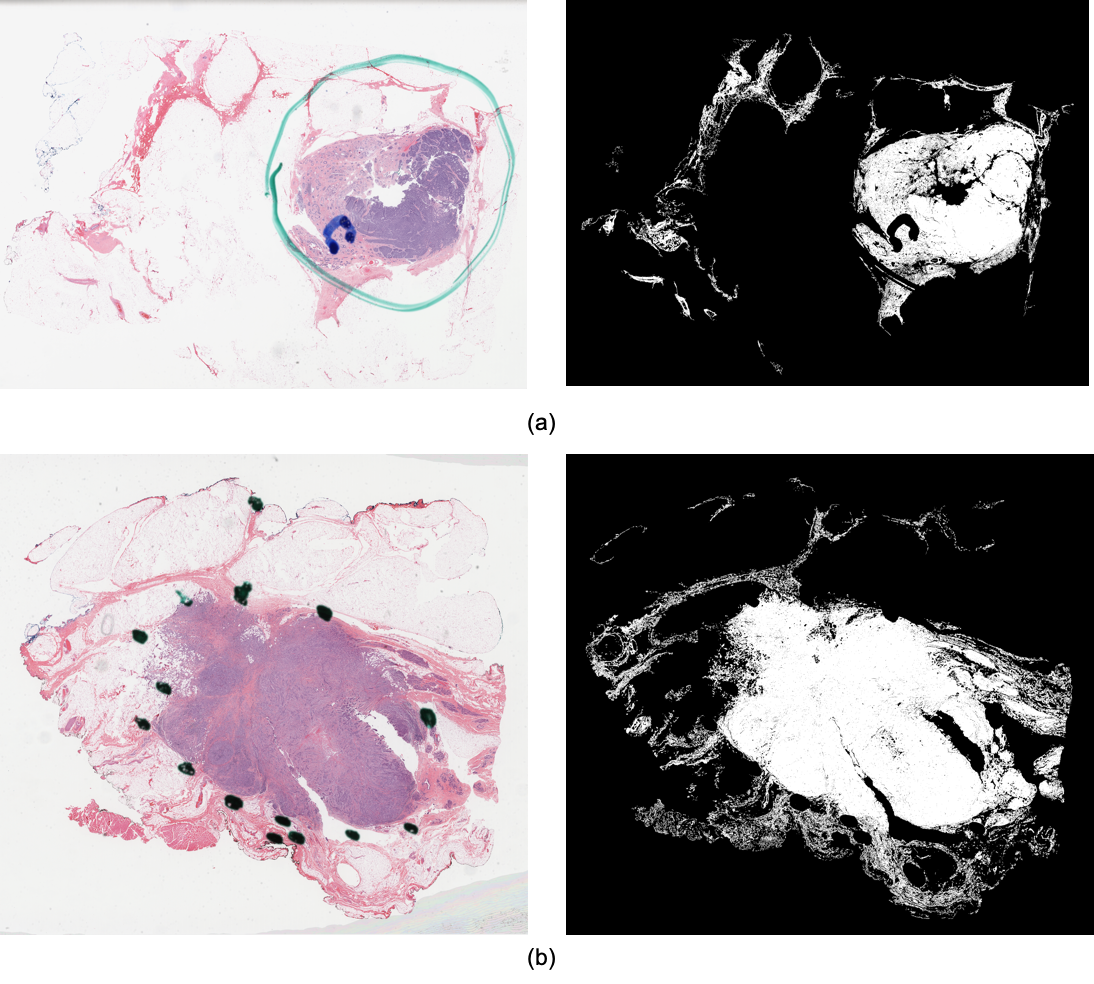


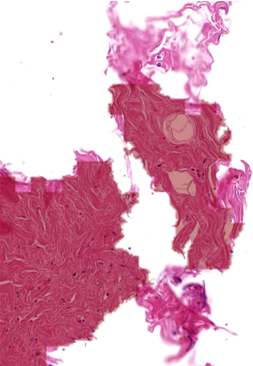

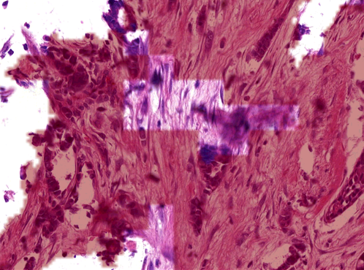


(c)

**Figure S12.** Artefact detection during ImageQC. (a) and (b) showing pen marking on a WSIs and its artefact-free mask. (c) showing artefact-free area in red shade, non-shaded regions showing blurriness (with blue arrows) and pen marking (with black arrows).

### **S3.9.2 Annotation Quality analysis**

Figure S13 shows the count and area of different region annotations. Some regions require small/detained and many annotations because they can be found dispersed in many areas instead of big localised chunks. For example, the annotation count for stroma tumour associated is 11050 but the total area covered is about 200 mm^2^. On the other hand DCIS annotation count is 2714 but they cover the highest area of about 400 mm^2^. Figure S14 shows inter-annotator agreement. It can be observed from Figure S14 that though some regions such as fibrocystic change and lobular neoplasia are relatively low in terms of annotated area (0.674 mm^2^ and 0.539 mm^2^, respectively), agreement on them is still very good (> 80%). Similarly, for some regions it is very hard for two annotators to annotate the exact regions because of no clear boundaries such as stroma tumour associated and hence a low agreement (about 44%).


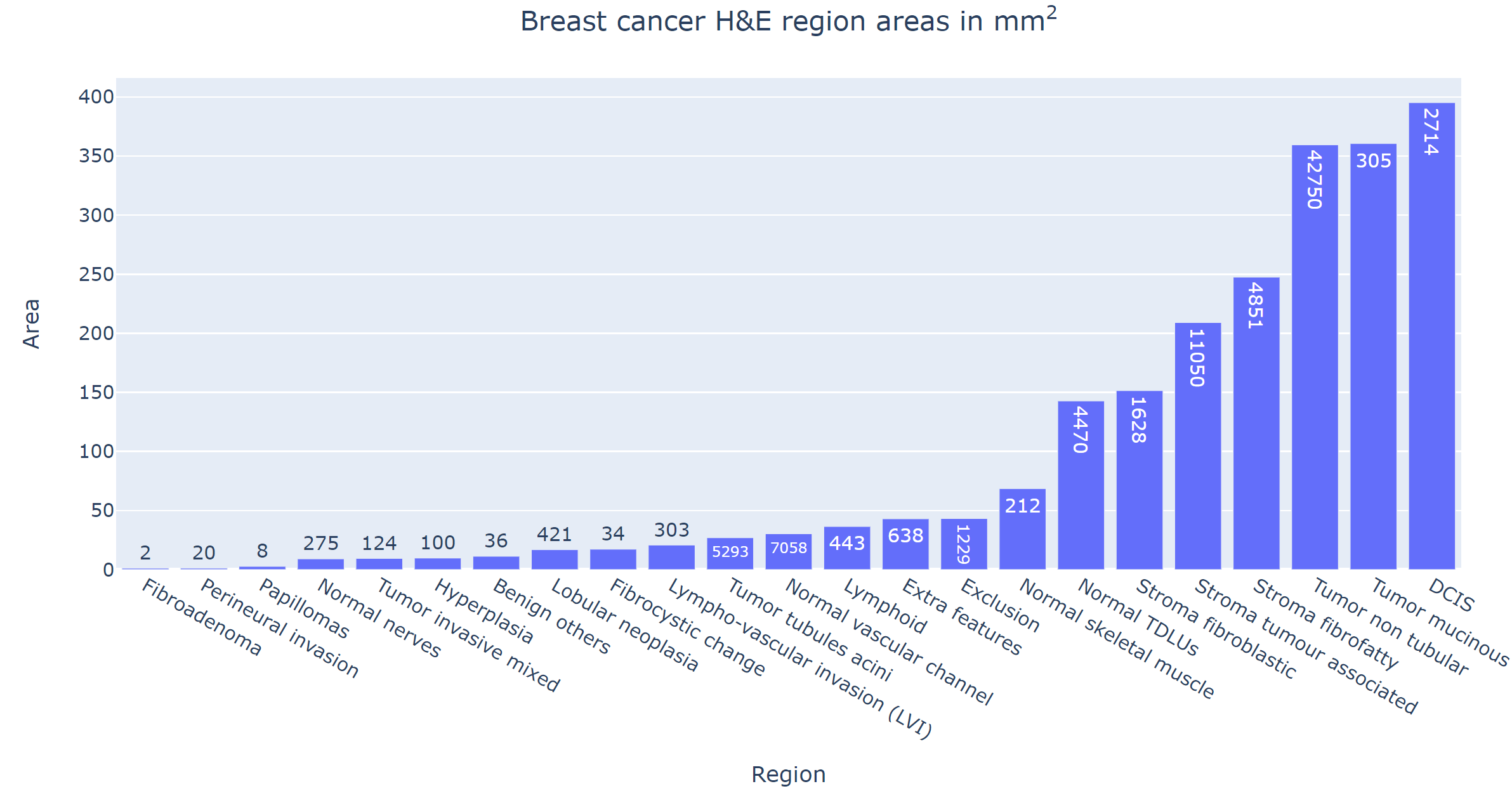


**Figure S13.** Breast H&E region annotation count and area for 314 cases. The numbers on the bars denote the number of regions annotated whereas the numbers on the y-axis represent the area in mm^2^.


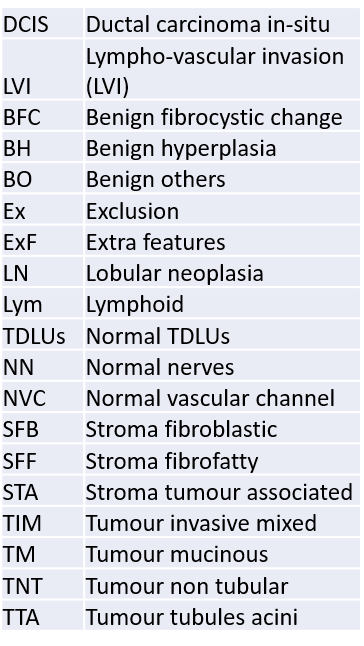

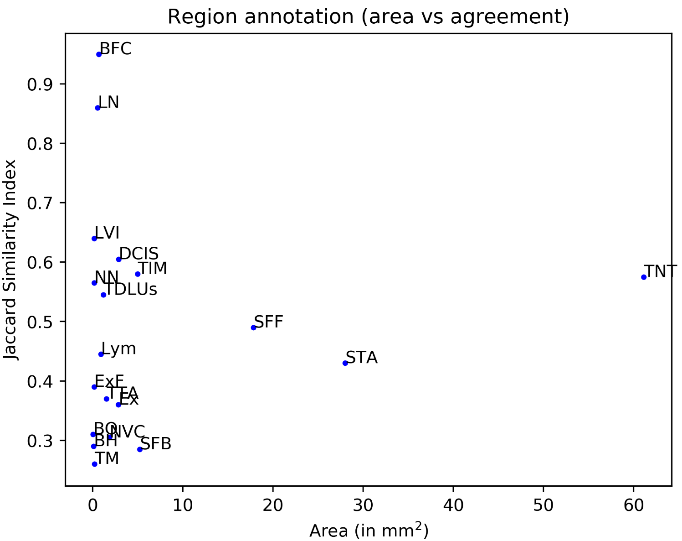


**Figure S14.** Inter-pathologist agreement on sample regions of breast H&E images. x-axis shows the area in mm^2^ whereas y-axis shows Jaccard Similarity Index.

### **S3.9.3 Pathologist agreement**

**Table S14.** Confusion matrix of different types of breast H&E cell annotation by two pathologists

|  |  | Pathologist 2 | | | | | | |
| --- | --- | --- | --- | --- | --- | --- | --- | --- |
|  | **Cell type** | **Mitosis** | **Normal epithelial** | **Stroma** | **TILs** | **Tumour NP1** | **Tumour NP2** | **Tumour NP3** |
| Pathologist 1 | Mitosis | **43** | 0 | 2 | 0 | 6 | 0 | 0 |
|  | Normal epithelial | 0 | **318** | 61 | 0 | 1 | 146 | 5 |
|  | Stroma | 0 | 0 | **2,852** | 0 | 530 | 397 | 9 |
|  | TILs | 1 | 26 | 469 | **1,600** | 162 | 38 | 5 |
|  | Tumour NP1 | 0 | 0 | 0 | 0 | **4,360** | 4 699 | 352 |
|  | Tumour NP2 | 0 | 0 | 0 | 0 | 0 | **1,353** | 638 |
|  | Tumour NP3 | 0 | 0 | 0 | 0 | 0 | 0 | **207** |

### **S3.9.4 Annotation usage**

Table S15 list three-folds cross-validation classification performance of HoVer-Net when fine-tuned with the cell-level annotations. The ‘fine-tuning’ strategy on the BraCe annotation was to keep the encoder weights frozen while the weights for the decoders for segmentation and classification were trained with a learning rate of 0.0001 for the first 10 epochs and 0.00001 for the next 20 epochs.

**Table S15.** Using cell-level annotation for machine learning. Pr (precision), Re (recall)

| Model | Overall (Macro-avg) | | | Tumour | | | TILs | | | Stromal | | | Norm-epithelial | | |
| --- | --- | --- | --- | --- | --- | --- | --- | --- | --- | --- | --- | --- | --- | --- | --- |
|  | Pr | Re | F1 | Pr | Re | F1 | Pr | Re | F1 | Pr | Re | F1 | Pr | Re | F1 |
| *M1 | 0.69±0.05 | 0.52±0.08 | **0.55**±0.08 | 0.75±0.02 | 0.95±0.02 | **0.83**±0.01 | 0.94±0.03 | 0.57±0.11 | **0.71**±0.07 | 0.88±0.06 | 0.32±0.06 | **0.47**±0.07 | 0.18±0.19 | 0.24±0.17 | **0.20**±0.18 |
| *M2 | 0.82±0.09 | 0.79±0.02 | **0.79**±0.07 | 0.92±0.01 | 0.92±0.04 | **0.92**±0.02 | 0.92±0.04 | 0.74±0.11 | **0.82**±0.06 | 0.81±0.04 | 0.81±0.03 | **0.81**±0.03 | 0.60±0.33 | 0.69±0.08 | **0.59**±0.22 |

*M1: Pretrained HoVer-Net

*M2: Fine-tuned HoVer-Net
